# A requirement for Krüppel Like Factor‐4 in the maintenance of endothelial cell quiescence

**DOI:** 10.3389/fcell.2022.1003028

**Published:** 2022-11-08

**Authors:** Victoria Mastej, Cassondra Axen, Anita Wary, Richard D. Minshall, Kishore K. Wary

**Affiliations:** Department of Pharmacology and Regenerative Medicine, University of Illinois, Chicago, IL, United States

**Keywords:** endothelial cells, EndMT, fibrosis, transcription factor, quiescence, KLF2, KLF4, VEGF

## Abstract

**Rationale and Goal:** Endothelial cells (ECs) are quiescent and critical for maintaining homeostatic functions of the mature vascular system, while disruption of quiescence is at the heart of endothelial to mesenchymal transition (EndMT) and tumor angiogenesis. Here, we addressed the hypothesis that KLF4 maintains the EC quiescence.

**Methods and Results:** In ECs, KLF4 bound to KLF2, and the KLF4-transctivation domain (TAD) interacted directly with KLF2. KLF4-depletion increased KLF2 expression, accompanied by phosphorylation of SMAD3, increased expression of alpha-smooth muscle actin (αSMA), VCAM-1, TGF-β1, and ACE2, but decreased VE-cadherin expression. In the absence of Klf4, Klf2 bound to the *Klf2*-promoter/enhancer region and autoregulated its own expression. Loss of EC-*Klf4* in *Rosa* ^*mT/mG*^ *::Klf4* ^*fl/fl*^ *::Cdh5* ^*CreERT2*^ engineered mice, increased Klf2 levels and these cells underwent EndMT. Importantly, these mice harboring EndMT was also accompanied by lung inflammation, disruption of lung alveolar architecture, and pulmonary fibrosis.

**Conclusion:** In quiescent ECs, KLF2 and KLF4 partnered to regulate a combinatorial mechanism. The loss of KLF4 disrupted this combinatorial mechanism, thereby upregulating KLF2 as an adaptive response. However, increased KLF2 expression overdrives for the loss of KLF4, giving rise to an EndMT phenotype.

## Highlights

• Adult endothelial cells (ECs) are quiescent in that these cells are arrested at G_0_-phase of the cell cycle, but mechanisms of EC quiescence are not well understood.
• The Krüppel-like factors (KLFs) -2 and -4 are transcriptional regulators, highly expressed in quiescent ECs, however, their roles in this process have not been addressed.
• Elucidation of the mechanisms of KLF function in quiescent ECs should provide clues to the translational discoveries intended for the treatment of EC-dysfunction, such as endothelial to mesenchymal transition (EndMT) associated with several vascular diseases including tumor angiogenesis.

## 1 Introduction

In the adult, ECs are highly specialized, heterogenous, and form a continuous monolayer via cell-cell and cell-matrix interactions (Aird, 2007; Eilken and Adams, 2010; Giannotta et al., 2013; Komarova et al., 2017). These interactions are the defining hallmarks of EC quiescence (Carmeliet et al., 2009; De Smet et al., 2009; Komarova et al., 2017; Schlereth et al., 2018; Ricard et al., 2021). EC quiescence is regarded as the default cellular state for a mature EC monolayer (Aird, 2007; De Smet et al., 2009; Komarova et al., 2017; Schlereth et al., 2018; Ricard et al., 2021). Quiescent ECs critically regulate the anti-adhesive, anti-inflammatory, and anti-thrombotic behavior of quiescent blood vessels. Conversely, the disruption of EC quiescence in response to regenerative stimuli or injury from virus, bacteria, or angiogenic factors are likely to be at the heart of EC dysfunction, EndMT, systemic sclerosis (SSc, or scleroderma) including tumor angiogenesis (Takabatake et al., 2005; Zeisberg et al., 2007a; Manetti et al., 2010; Cooley et al., 2014; Chen et al., 2015; Batlle et al., 2019; Bischoff, 2019; Chen et al., 2019; Yao et al., 2019; Eapen et al., 2020; Rossato et al., 2020). Importantly, EndMT is associated with the development of resistance to anti-cancer therapy, cardiotoxic effects of anti-cancer drugs; however, the molecular mechanisms of EndMT are not completely understood.

Hypoxia and tumor angiogenic factors, such as Vascular Endothelial Growth Factor (VEGF) can activate and transform quiescent ECs into proliferative stalk cells, this event propagates signaling that results in EndMT, produce *de novo* fibroblast and myofibroblast activation, extracellular matrix (ECM) deposition, and tissue fibrosis (Bischoff, 2019; Chen et al., 2019; Yao et al., 2019; Eapen et al., 2020; Rossato et al., 2020). Analogous to epithelial to mesenchymal transition (EMT) (Lamouille et al., 2014), EndMT is associated with the loss of VE-cadherin localized in adherens junctions, increased expression of mesenchymal transcription factors, loss of cellular polarity, and activation of TGF-β signaling frequently seen in melanoma and Kaposi sarcoma (Munger et al., 1999; Zeisberg et al., 2007b; Gasperini et al., 2012; Li et al., 2018). Deletion of EC transcription factor, such as *Twist* and *Snail,* inhibited EndMT and ameliorated kidney fibrosis in mouse model (Lovisa et al., 2020). Silencing of epigenetic modifier JMJD2B in ECs reduced TGF-β2-induced expression of mesenchymal genes, limited the extent of EndMT (Glaser et al., 2020). Thus, chronic inflammation and immune cell dysfunction are associated with EndMT (Munger et al., 1999; Zeisberg et al., 2007b; Gasperini et al., 2012; Li et al., 2018; Glaser et al., 2020; Lovisa et al., 2020). The loss of quiescent phenotype and the acquisition of EndMT are also associated with pulmonary arterial hypertension (Carmeliet et al., 2009; De Smet et al., 2009; Shatat et al., 2014a; Stenmark et al., 2016; Komarova et al., 2017; Ranchoux et al., 2018). Thus, chronic inflammation and sustained release of cytokines propagate signaling crosstalk, release of proteases, degradation of ECM components are considered hallmarks of EndMT and tumor microenvironment. However, the mechanisms of these processes are incompletely understood.

Transcription factors (TFs) KLF2 and KLF4 are highly expressed in quiescent ECs (Kuo et al., 1997; Hamik et al., 2007; Cowan et al., 2010; Zhou et al., 2012; Sangwung et al., 2017; Denis et al., 2019; Han et al., 2021). To this end, KLF4 is known to inhibit cell cycle progression, while KLF2 inhibited VEGF-induced hyperpermeability. Importantly, KLF4 and KLF2 are expressed in differentiated endothelial and epithelial cell subpopulations (Zhang et al., 2000; Chen et al., 2001; Dekker et al., 2002; Katz et al., 2002; Bhattacharya et al., 2005; Zhou et al., 2012; Shatat et al., 2014b; Sangwung et al., 2017; Denis et al., 2019). Both KLF2 and KLF4 contain a DNA binding domain composed of conserved C2H2 zinc fingers, a transactivation and repressor domain, and a nuclear localization signal (Katz et al., 2002; Bhattacharya et al., 2005; Alaiti et al., 2012; Sangwung et al., 2017). Additionally, KLF2 and KLF4 both share common regulatory mechanisms in that they recruit p300-CBP coactivator protein (Shields and Yang, 1997; SenBanerjee et al., 2004; Swamynathan et al., 2011). Here, we addressed the hypothesis that EC-KLF4 and KLF2 maintain the quiescent state of ECs, while the loss of EC-KLF4 gives rise to EndMT.

## 2 Results

### 2.1 Expression of KLF4, KLF2, ACE2, and TGF-β1 associated with pulmonary microvessel remodeling in emphysema and COPD patients

KLF4 and KLF2 are abundantly expressed in normal adult lungs tissues (Supplementary Figure S1). The status of KLF2 and KLF4 expression in human chronic lung inflammatory disease have not been reported. To address this gap, lung tissue sections prepared from healthy control donors, emphysema and COPD patients were stained with anti-KLF2 or anti-KLF4 antibodies (Supplementary Figures S2A–D). Microscopic analyses of lung tissue sections showed thickening of alveolar structures in emphysema and COPD patients, compared with healthy vessels in donors (Supplementary Figures S2A–D). Quantification of KLF4 and KLF2 staining data in these tissue sections suggest that in normal lung, the level of KLF2 is relatively lower than KLF4 (Supplementary Figure S2E), in contrast, in emphysema and COPD tissue sections, the levels of KLF2 is higher than KLF4 (Supplementary Figure S2E). Total cell lysates prepared from a limited number of tissue samples, were subjected to Western blotting (WB) with antibodies against KLF4, KLF2, ACE2, TGF-β1, VE-cadherin and GAPDH. As VE-cadherin is exclusively expressed by mature ECs, we included VE-cadherin to demonstrate that presence of ECs in lung tissue samples we analyzed (Supplementary Figure S2F). We included TGF-β1, as previous studies have shown the ability of epithelial cell KLF4 to transcriptionally induce the expression of TGF-β1, thereby inducing epithelial to mesenchymal transition (EMT). Quantification of signal intensities of these proteins suggest that, the level of KLF4 is relatively higher in control lung tissues, while the expression of KLF2 increased and KLF4 decreased. Importantly, we detected increased ACE2 expression in lung disease tissue samples. As such, we also found evidence of EndMT in diseased lung tissue, as illustrated by co-alignment of anti-α-smooth muscle actin (αSMA) in CD31^+^ vascular structures (Supplementary Figure S3). Although, we surveyed limited number of tissue samples, together these data provided us the impetus to test the hypothesis that oscillation of KLF4 and KLF2 expression might be associated with EC-dysfunction and EndMT in the lung.

### 2.2 Oligomeric KLF4, KLF2, and p300 protein complexes in quiescent ECs

**FIGURE 1:**
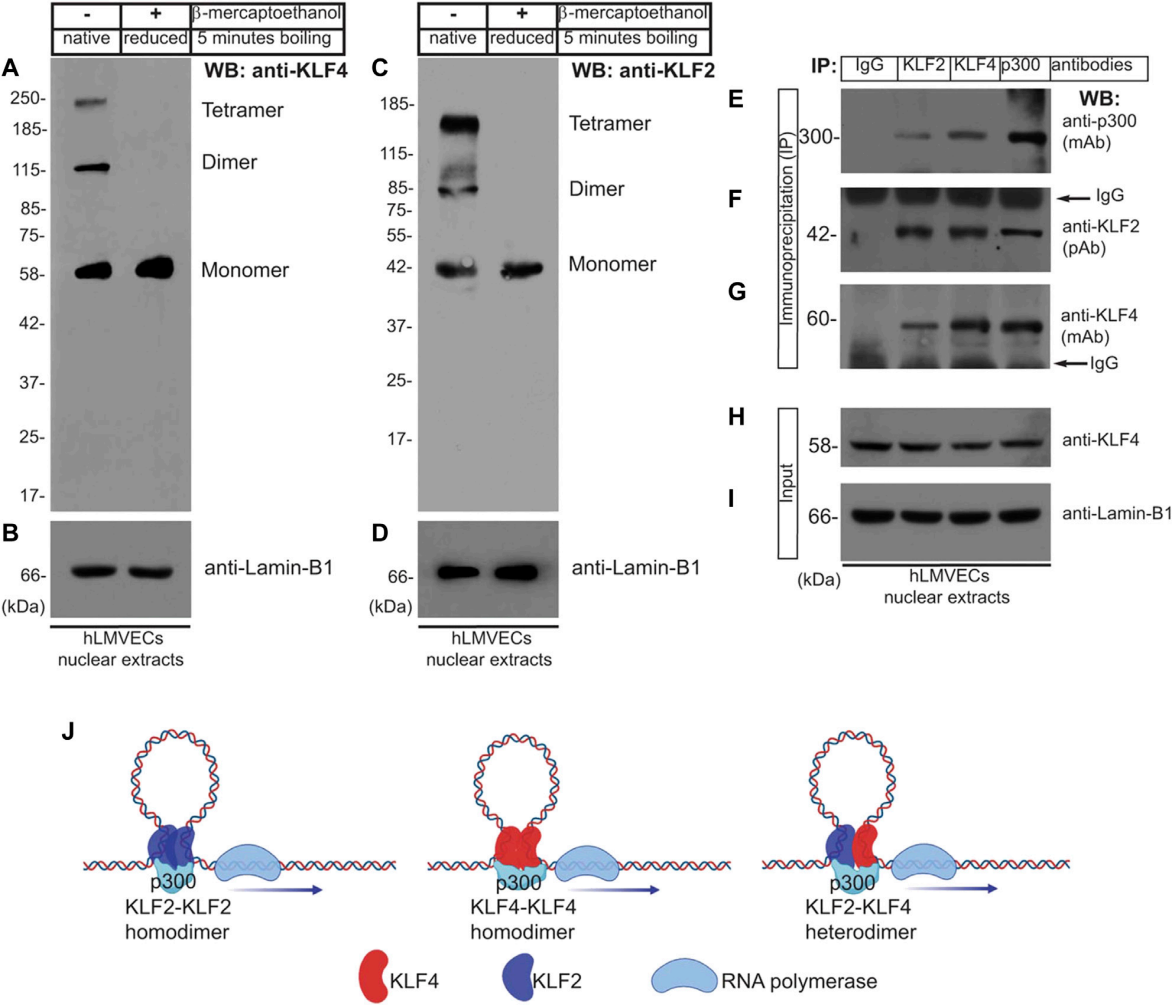
Oligomeric KLF4 and KLF2 protein complexes in quiescent ECs. **(A,C)** Nuclear extracts prepared from human lung microvascular endothelial cells (hLMVECs) were boiled in native or reducing sample buffers, resolved by 9% SDS-PAGE. The presence of mono-, di-, and tetrameric anti-KLF4 and anti-KLF2 polypeptide species in native SDS-PAGE were analyzed by indicated antibodies. **(B,D)** Equal loading were analyzed by anti-Lamin-B1 antibody. **(E–G)** hLMVECs extracts were subjected to immunoprecipitation (IP) with IgG (control), anti-KLF2, anti-KLF4, and anti-p300 antibodies, and analyzed by WB with indicated antibodies. **(H,I)** Nuclear lysates (input) were analyzed by WB with anti-KLF4 and anti-Lamin-B1 antibodies. Molecular weights are given in kiloDalton (kDa). This experiment was repeated at least three times. **(J)**. Models showing homodimer and heterodimer KLF2 and KLF4 protein complexes.

Most transcription factors form homo- or heterodimeric protein complexes. KLF2 and KLF4 have been shown to associate directly with p300, a nuclear protein that functions as a histone acetyltransferase (HAT) which regulates transcription via chromatin remodeling (Das et al., 2006; Feinberg et al., 2005; SenBanerjee et al., 2004; Shields and Yang, 1997; Swamynathan et al., 2011) but is not known to promote the formation of homo- or heterodimeric protein complexes. To test the hypothesis that KLF4 and KLF2 form multi-molecular complexes in human lung microvascular (hLMVECs), RIPA buffer solubilized nuclear lysates prepared from these cells were subjected to: 1) Western Blot (WB) analysis and 2) reciprocal coimmunoprecipitation experiment using anti-IgG (control), anti-KLF2, anti-KLF4, and anti-p300 antibodies, thereafter analyzed by WB immunoblotting with indicated antibodies. Equal amounts of proteins were boiled in native or reducing sample buffer and analyzed for occurrence of oligomeric polypeptide species (Figures 1A–D). We detected presence of mono-, di-, and tetrameric anti-KLF4 and anti-KLF2 polypeptide species by native SDS-PAGE whereas in presence of strong reducing conditions (5% β-mercaptoethanol), only monomeric KLF4 and KLF2 were detected (Figures 1A–D). Next, WB analysis was used to detect anti-p300 polypeptide immunoreactive species in anti-KLF2, anti-KLF4 and anti-p300 immunoprecipitated complexes (Figure 1E). WB analysis with anti-KLF2 antibody showed the presence of ∼42 kDa protein in anti-KLF4, anti-KLF2, and anti-p300 immunoprecipitates and anti-KLF4 antibody detected a ∼58 kDa signal in anti-KLF4, anti-KLF2, and anti-p300 immunecomplexes; control IgG did not precipitate any of the above polypeptide species (Figures 1E–G). To address equal loading across the lanes, total nuclear lysates (inputs) were analyzed by anti-KLF4 and anti-lamin-B1 antibodies (Figure 1H,I). Together these data show that KLF2, KLF4, and p300 likely form mono-, di- or an oligomeric protein complexes in hLMVEC quiescent ECs (Figure 1J).

### 2.3 Transactivation domain (TAD) of KLF4 binds to KLF2

**FIGURE 2:**
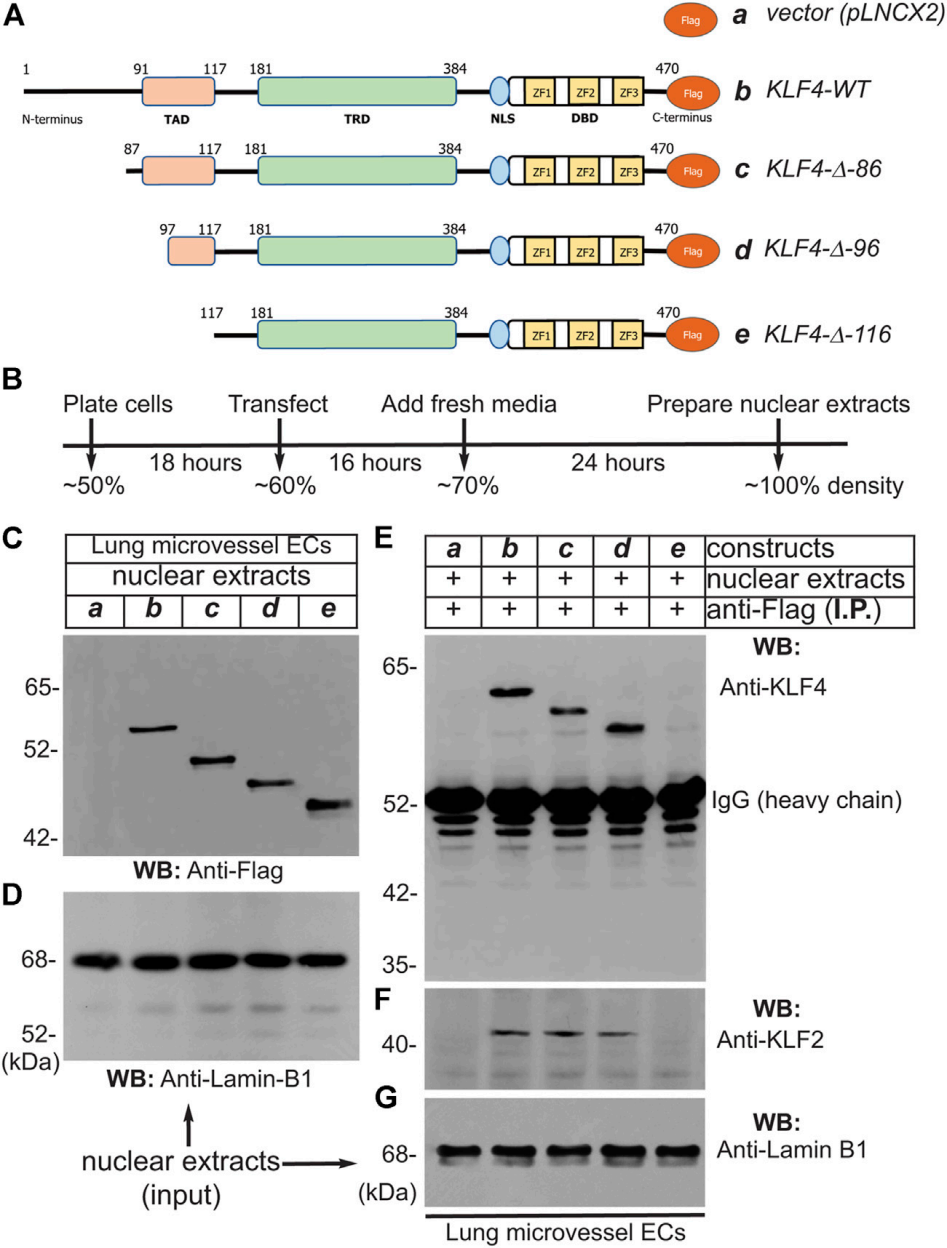
Transactivation domain (TAD) of KLF4 binds to KLF2. **(A)** pLNCX2 retroviral vector encoding: **(a)** vector alone; **(b)** human *KLF4-wild-type (WT) cDNA* fused in-frame with Flag-epitope tag; **(c)** *KLF4-Δ-86* construct (lacking 85 amino acid residues from the N-terminus) fused in-frame with Flag-epitope tag; **(d)** *KLF4-Δ-96* construct (lacking 95 amino acid residues from the N-terminus) fused in-frame with Flag-epitope tag; **(e)** *KLF4-Δ-116* construct (lacking 115 amino acid residues from the N-terminus) fused in-frame with Flag-epitope tag as shown. **(B)** Strategy and timeline of human lung microvessel endothelial cell (hLMVECs) infection/transfection experiments. **(C)** Efficiency of retrovirus infection of hLMVECs was determined by WB with anti-Flag antibody. **(D)** Equal loading across the lanes were judged by anti-Lamin-B1 antibody. **(E)** Nuclear cell extracts were immunoprecipitated with an anti-Flag monoclonal antibody (mAb) and analyzed by WB with anti-Flag mAb. **(F)** The *KLF4-wild-type, KLF4-Δ-86,* and *KLF4-Δ-96* immuneprecipitated KLF2 polypeptide species, while *KLF4-Δ-116* did not. **(G)** Equal levels checked with anti-Lamin-B1. These experiments were repeated at least 3 times.

To address which segment is responsible for interaction of KLF4 with KLF2, we engineered a series of retroviral constructs encoding human KLF4-cDNA wild-type and deletion mutants, performed transfections, and conducted immunoprecipitation experiments using Flag-epitopes (-DYKDDDDK-, 1,012 Da) that were fused in-frame to the C-terminus (Figure 2A). The methods and efficiency of lentivirus vector pLNCX2-mediated transfection have been previously described by us (Humtsoe et al., 2003; Humtsoe et al., 2010). The strategy and timeline of transfection of pLNCX2 retroviral constructs are shown in Figure 2B hLMVECs at 50% confluence were used for this set of experiments. Nuclear extracts prepared from hLMVECs expressing 1) pLNCX2 vector alone, 2) human KLF4-WT cDNA, 3) KLF4-Δ-86 construct (lacking 85 amino acid residues from the N-terminus), 4) KLF4-Δ-96 construct (lacking 95 amino acid residues from the N-terminus), or 5) KLF4-Δ-116 construct (lacking 115 amino acid residues from the N-terminus) and subjected to WB analyses showed the presence of corresponding polypeptide species (Figure 2C). Equal loading was determined by WB analyses with anti-lamin-B1 antibody (Figure 2D). Next, nuclear cell extracts that were immunoprecipitated with anti-Flag protein antibody were immunoblotted with anti-KLF4 antibody revealed bands for KLF4-WT (b), KLF4-Δ-86 (c), and KLF4-Δ-96 (d), while in control and KLF4-Δ-116 (e) samples, no band were detected (Figure 2E). The non-specific signal at ∼52 kDa represents IgG (heavy chain). Stripping and re-probing the WB shown in Figure 2E with anti-KLF2 Ab showed presence of KLF2 (Figure 2F). Figure 2G shows that an equal amount of nuclear extract was loaded in each lane (input). This data suggests that KLF4 likely binds to KLF2 via amino acid residues 97–117 present within the KLF4 transactivation domain (TAD).

**FIGURE 3:**
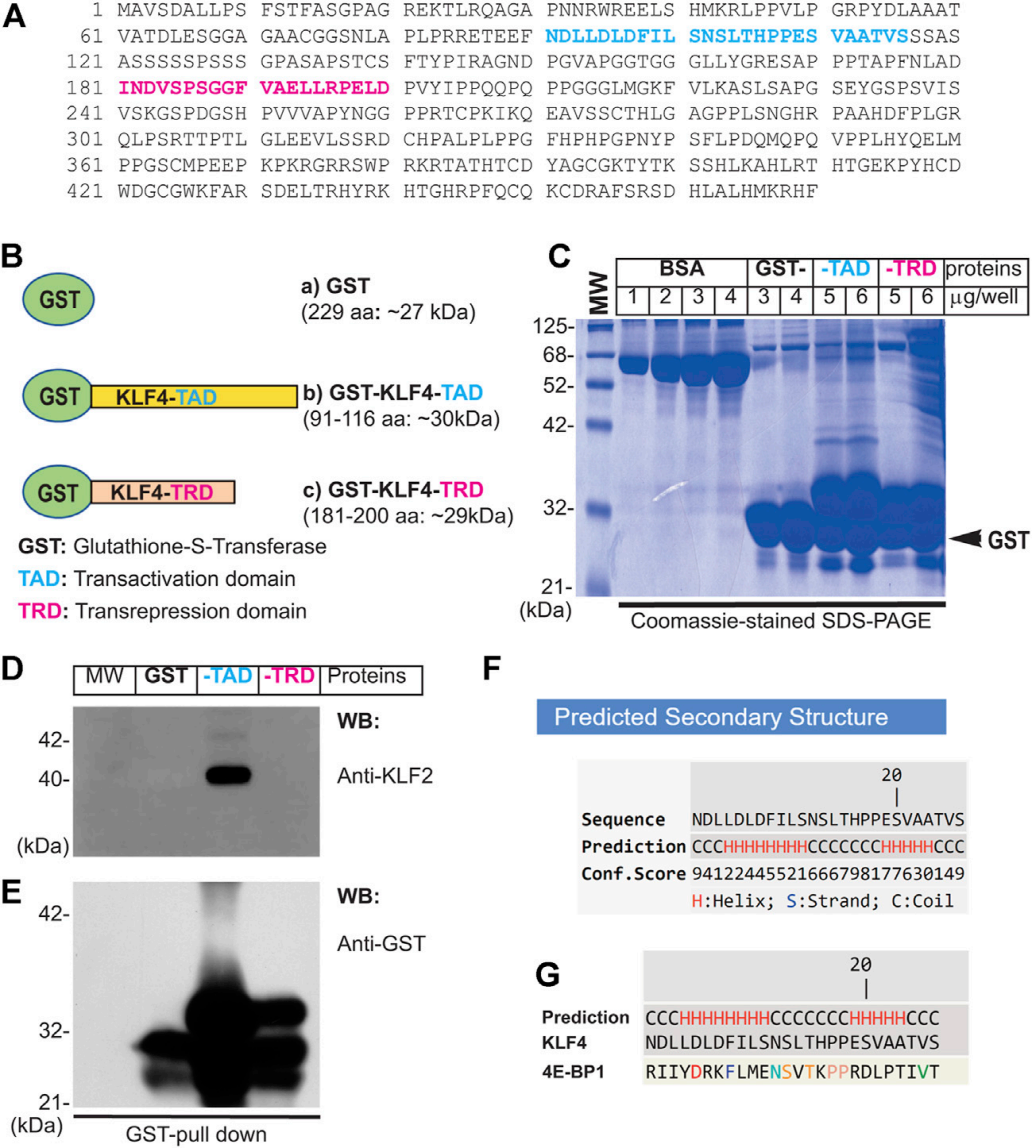
Direct interaction of KLF4 with KLF2. **(A)** Amino acid sequence of human KLF4. The amino acid (TAD) sequence 91–116 is shown in blue color, while magenta represent TRD region used for preparation of GST-fusion proteins. **(B)** Schematics of GST-fusion protein constructs: **(a)** GST-alone (∼27 kDa), **(b)** GST-KLF4-TAD (∼30 kDa), and **(c)** GST-KLF4-TRD (∼29 kDa). **(C)** GST-fusion proteins were expressed in *E coli* (BL2) and purified. **(C)** The integrity of GST-fusion proteins. **(D)** hLMECs nuclear extracts were incubated with indicated sepharose-4B bound-GST-fusion proteins at 4^°^C, washed 5 times with nuclear extraction buffer, and resolved by SDS-PAGE. The NC membrane was probed with anti-KLF2 antibody. **(E)**. The membrane was stripped and reprobed with anti-GST antibody to examine equal loading of GST-fusion proteins across the lanes. Experiments were performed at least 3 times. **(F,G)** KLF4-TAD amino acid sequence, showing helix and coiled-coil secondary structures performed using I-TASSER online computational analytical tools.

Next, to address if the interaction of KLF4 and KLF2 is direct or indirect, we carried-out GST pull-down experiments (Figure 3). The amino acid sequence of human KLF4 (wild-type full-length) is shown in Figure 3A. The GST-cDNA was fused in-frame to the C-terminus of the human *KLF4*-*cDNA* segment encoding amino acid sequence 91–116 (TAD), shown in blue (construct-b), and to region 181–200 (TRD), shown in magenta (construct-c). These cDNA constructs were used for preparation of GST-fusion proteins expressed in protease-deficient *E. coli* (BL2), thereafter purified by Sepharose-4B column chromatography (Figure 3B&C). Next, nuclear extracts prepared from hLMVECs were used for GST-pull down experiments. The NC-membrane analyzed by WB showed the presence of expected anti-KLF2 antibody reactive ∼38 kDa polypeptide species (Figure 3D). The amount of GST-fusion protein in each sample was determined by anti-GST antibody to insure equal loading (Figure 3E). This data shows that KLF4-TAD protein interacts with KLF2, while KLF4-TRD segment did not. In addition, KLF4-TAD amino acid sequence (aa 91–117) was subjected to Iterative Threading ASSEmbly Refinement (I-TASSER) computational analysis. I-TASSER is a hierarchical method to protein structure prediction and structure-based function annotation (Yang et al., 2015; Yang and Zhang, 2015). Accordingly, prediction revealed that amino acid residues 91–117 in KLF4 contain two discrete α-helical domains and two short stretches of coiled-coil regions (Figures 3F,G). Further homology search identified 4E-BP1 that is structurally homologous to the KLF4 aa 91–117 secondary structure. 4E-BP1 is a translational repressor protein that binds to eukaryotic initiation factor 4E (eIF4E) and represses protein translation by inhibiting the ability of eIF4E to recruit 40S ribosomal subunits, for details see computational analyses in supplement section (Supplementary Figure S4). Thus, we identified a new protein interacting domain within KLF4-TAD that has an intrinsic ability to form homodimer, heterodimer and oligomeric protein complexes.

### 2.4 Endothelial *Klf4*-knockdown promotes upregulation of Klf2

**FIGURE 4:**
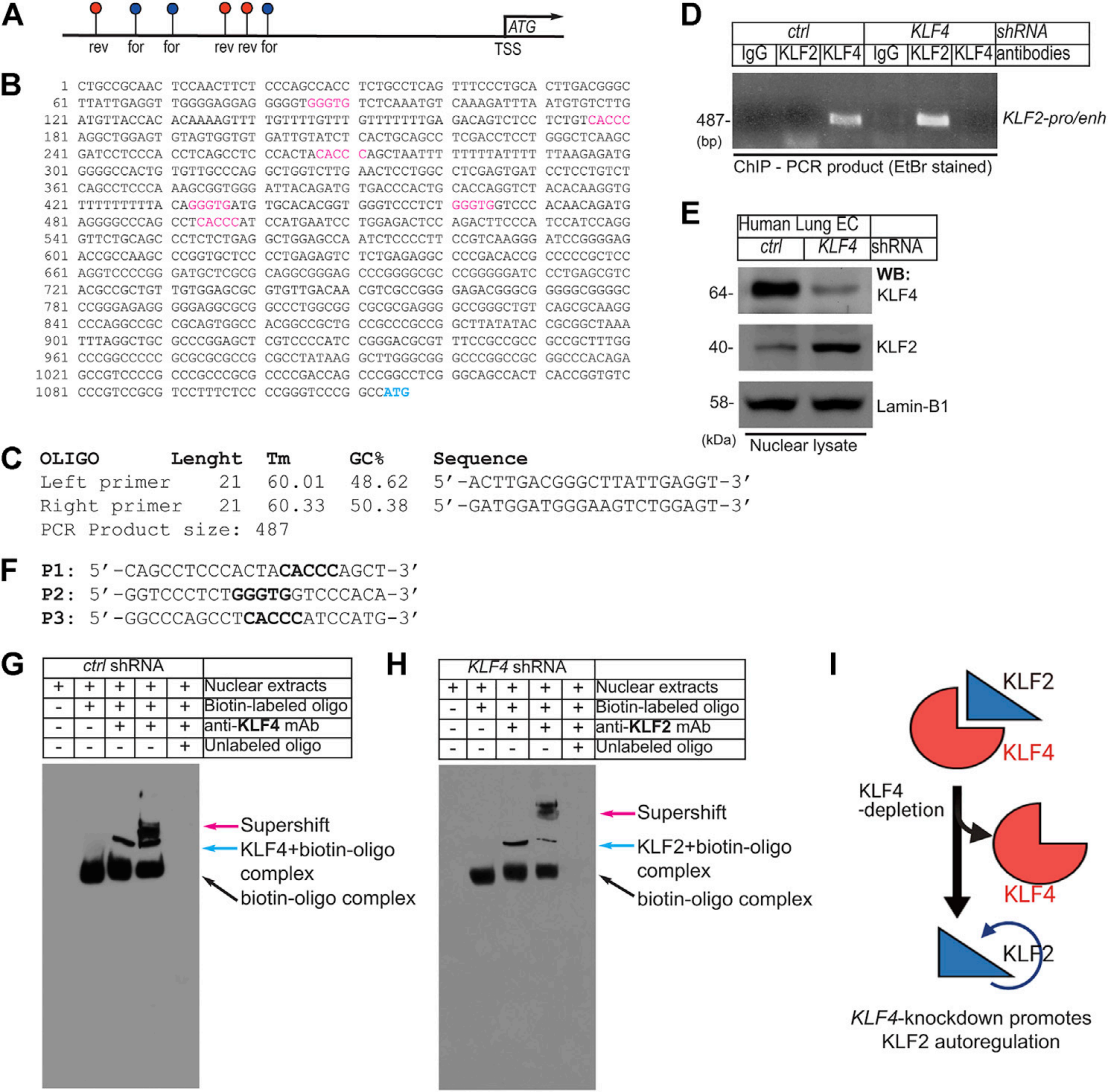
EC Klf4-depletion promotes Klf2 autoregulation. **(A)**. Schematics of ∼1.0 kbp upstream of the human *KLF2*-promoter/enhancer shows presence of 6 putative KLF4/KLF2 binding sites (blue, in forward-; red, in reverse-orientation). **(B)** DNA sequence of the human *KLF2*-promoter/enhancer segment. The putative KLF4/KLF2 binding sites are as shown (For-CACCC and Rev-GGGTG). ATG indicates transcription start site (TSS). **(C)** Oligonucleotide PCR-primer sequences used for amplifying KLF4/KLF2 binding sites. The PCR primers produce ∼487 bp PCR-product. **(D)** Chromatins were prepared after *control-shRNA* and *KLF4-shRNA* knockdown. Chromatins were immunoprecipitated with indicated antibodies and eluants were PCR-amplified by primers. The PCR-product indicates binding of KLF4 to cognate promoter/enhancer region harboring -CACCC- and -GGGTG- in the KLF2 promoter. In hLMVECs, subjected to *KLF4-shRNA,* KLF2 bound to its own promoter. **(E)** Efficiency of shRNA mediated knockdown are as shown. **(F)** Biotin-labeled oligonucleotides (P1, P2, and P3) designed after the *KLF2*-promoter DNA sequence used for EMSA. **(G, H)** Biotin-labeled oligonucleotide (P1) harboring the sequence (CACCC) of the putative KLF2/KLF4 binding sites designed after the *KLF2*-promoter were incubated with nuclear extracts. The supershift was carried-out by preincubation with excess nuclear extracts, prepared from **(G)** control-shRNA or **(H)** *KLF4*-shRNA knockdown ECs with indicated antibodies. Experiments were repeated 3 times. **(I)** Summary model showing the loss of KLF4 promoted binding of KLF2 to the *KLF2-*promoter/enhancer DNA sequence.

Close examination of ∼1.0 kbp upstream of the transcription start site (TSS) in the human *KLF2*-promoter/enhancer showed the presence of six putative KLF4/KLF2 binding sites, 5′-CACCC-3′ in forward and 5′-GGGTG-3′ in reverse orientation (Figure 4A). The DNA sequence of the human *KLF2*-promoter/enhancer segment is shown in Figure 4B. We thus designed oligonucleotide PCR-primers to amplify the flanking six putative KLF4/KLF2 binding sites on the *KLF2*-promoter DNA sequence (Figure 4C). Then, hLMVECs at 70–80% confluence were treated with *control-shRNA* and *KLF4-shRNA* to induce knockdown. Thereafter, sheared chromatins were immunoprecipitated with indicated antibodies and eluants were PCR-amplified (Figure 4D). The primers amplified a 487 bp PCR-product suggesting three was binding of KLF4 to the *KLF2*-promoter, while IgG and KLF2 did not. However, in *KLF4-shRNA* treated hLMVECs, KLF2 bound to its own promoter (middle lane), while IgG and KLF4 did not (Figure 4D). The efficiency of knockdown was analyzed by WB with indicated antibodies (Figure 4E). This data suggests that at homeostatic levels, KLF4 binds to the *KLF2* promoter, while loss or decreased expression of KLF4 allows KLF2 to auto-regulate its own expression by binding to its own promoter. To confirm this hypothesis, we performed an electrophoretic mobility shift assay (EMSA). Biotin-labeled oligonucleotide probes (P1, P2, and P3) designed based on the *KLF2*-promoter harboring 5′-CACCC-3′ or 5′-GGGTG-3′ DNA sequences were used for the EMSA. After incubation with nuclear extracts, biotin-labeled oligonucleotide probe interaction was observed between KLF4 with P1, with the super-shifts indicated by colored arrows (Figure 4G). In a converse experiment, EMSA was performed using KLF4-depleted nuclear extracts (Figure 4H); the result showed that in absence of KLF4, KLF2 binds to the same oligonucleotide probe (P1), indicating KLF4 and KLF2 are able to occupy the same exact DNA sequence.

### 2.5 *Klf4*-deletion in quiescent ECs propagates an EndMT phenotype

**FIGURE 5:**
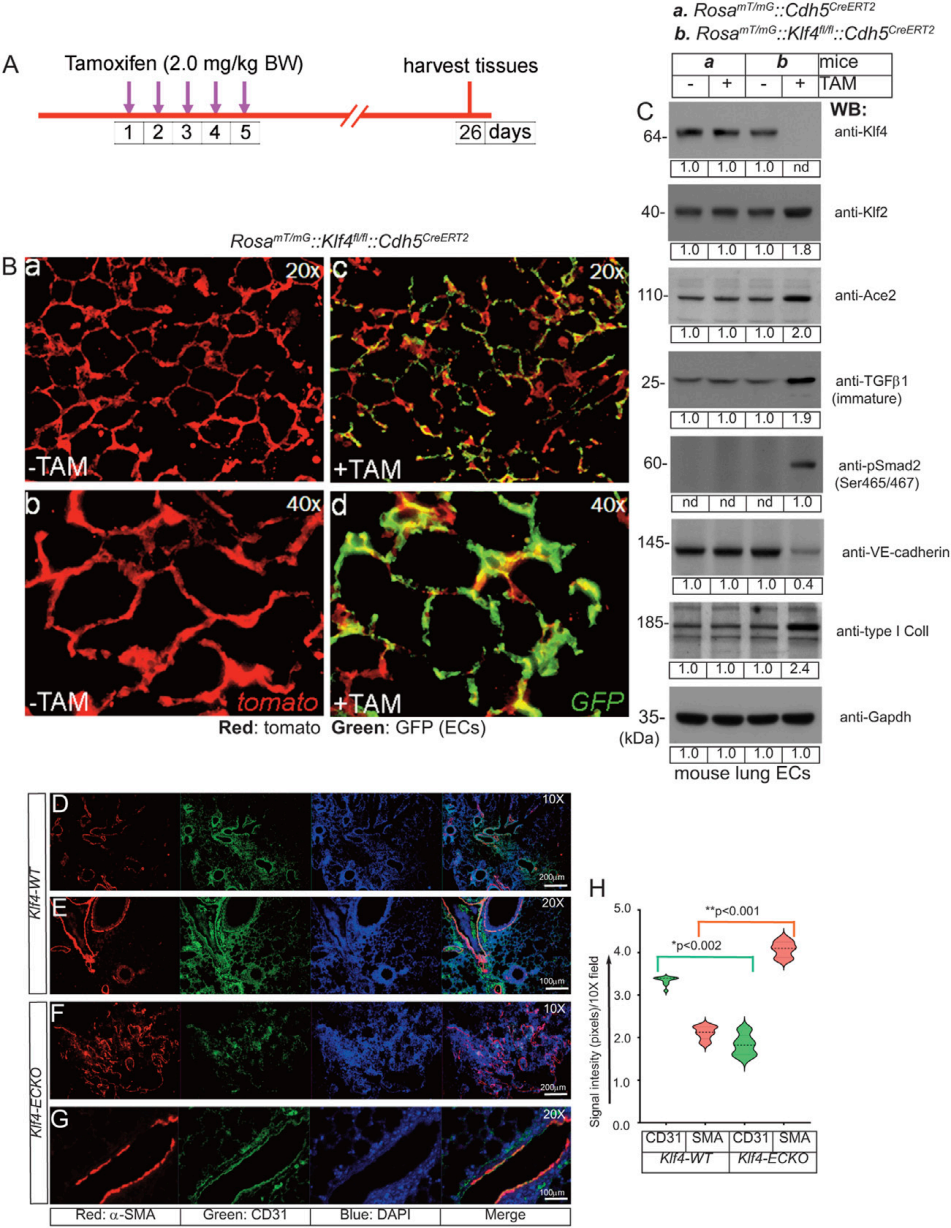
EC-*Klf4* deletion induces EndMT in the lung tissues. **(A)** The timeline and TAM treatment scheme; **(B)** Mice are injected with TAM for five consecutive days to induce deletion of EC-*Klf4* alleles and allow EC-GFP expression. In absence of TAM, all cells in reporter mouse express tomato (red) fluorescence. Lung tissue samples are harvested on day 26. Magnifications are as shown. **(C)** EC extracts were analyzed by WB with indicated antibodies. Molecular weights are given in kiloDalton (kDa). The numbers below each WB panel indicate signal quantification. Experiments were performed at least 3 times. **(D–G)** Immunohistological analysis of lung tissues prepared from *Klf4-WT* (-TAM) and *Klf4-ECKO* (+TAM) mice (*Klf4-WT*, n = 10; *Klf4-ECKO*, *n* = 10) with anti-CD31 and anti-α-SMA. Note that increased anti-α-SMA antibody immunoreactivities (red) exclusively co-aligned with CD31^+^ vascular structures. Nuclei were counterstained with DAPI (blue). Magnification of images are as shown. Experiments are done in both male and female mice (5 + 5) and the EndMT pathology were identical. Magnifications and scale bars are as shown. **(H)** Quantification of red (SMA) and green (CD31) signal intensities using NIH ImageJ software in arbitrary units; *p < 0.002; **p < 0.001 compared with Klf4-WT; unpaired 2-tailed Student’s t test. At least eight 10X microscopic fields/per slide were used for quantification and 5 slides were used for each group. Staining experiments were repeated at least 3 times and examined under Olympus Epifluorescence microscope in room temperature. Images were saved as raw data, converted into TIFF format and combined using QuarkXpress 2020 software, finally saved as TIFF documents.

To address the *in vivo* role of KLF4 in EC quiescence, we bred and crossbred several different strains of mice to produce: *Klf4* ^*fl/fl*^ *::Cdh5* ^*CreERT2*^ and (Eilken and Adams, 2010) *Rosa* ^*mT/mG*^ *::Klf4* ^*fl/fl*^ *::Cdh5* ^*CreERT2*^ mice lines in C57BL background (Supplementary Figure S4A−D). The *Cdh5*-promoter driven CreERT2-mediated genetic deletion following TAM administration allowed EC-lineage tracing (Figure 5A,B). Next, CD45^−^and CD31^+^ ECs (see methods) from the control and TAM-administered mice were used for biochemical analyses. Accordingly, WB analyses of cell extracts prepared from CD45^−^and CD31^+^ lung ECs isolated from these mice confirmed Klf4-silencing (henceforth called *Klf4* ^ECKO^). In *Klf4* ^*ECKO*^ mice, we observed increased Klf2 expression (Figure 5C), increased TGF-β1, increased type-I collagen protein levels, and phosphorylation of Smad3 (Ser465/467), but decreased VE-cadherin (Figure 5C). Together, these data strongly suggest that *Klf4*-silencing is associated with increased Klf2-expression and EndMT.

To further address the underlying relationship between the loss of quiescent EC-Klf4 and EndMT of lung ECs, we monitored the effect of *Klf4*-deletion in *Klf4* ^ECKO^ mice (Figures 5D–H). Four weeks after the last TAM injection, microscopic examination of histological tissue sections showed a profound increase in anti-αSMA antibody immunoreactivity of CD31^+^ vascular structures in *Klf4-ECKO* lung tissues (Figures 5F, G). Quantification showed a >50% decrease in CD31^+^ signal intensity (Figure 5H), while anti-αSMA immunoreactivity increased significantly (Figure 5H). These data indicate that in *Klf4*-ECKO lung tissues, ECs acquire the *bona fide* mesenchymal marker α-SMA, consistent with the WB data shown in Figure 5C indicating EC-*Klf4*-silencing is strongly associated with EndMT in *Klf4*-ECKO.

### 2.6 *Klf4*-deletion in ECs leads to pulmonary fibrosis

**FIGURE 6:**
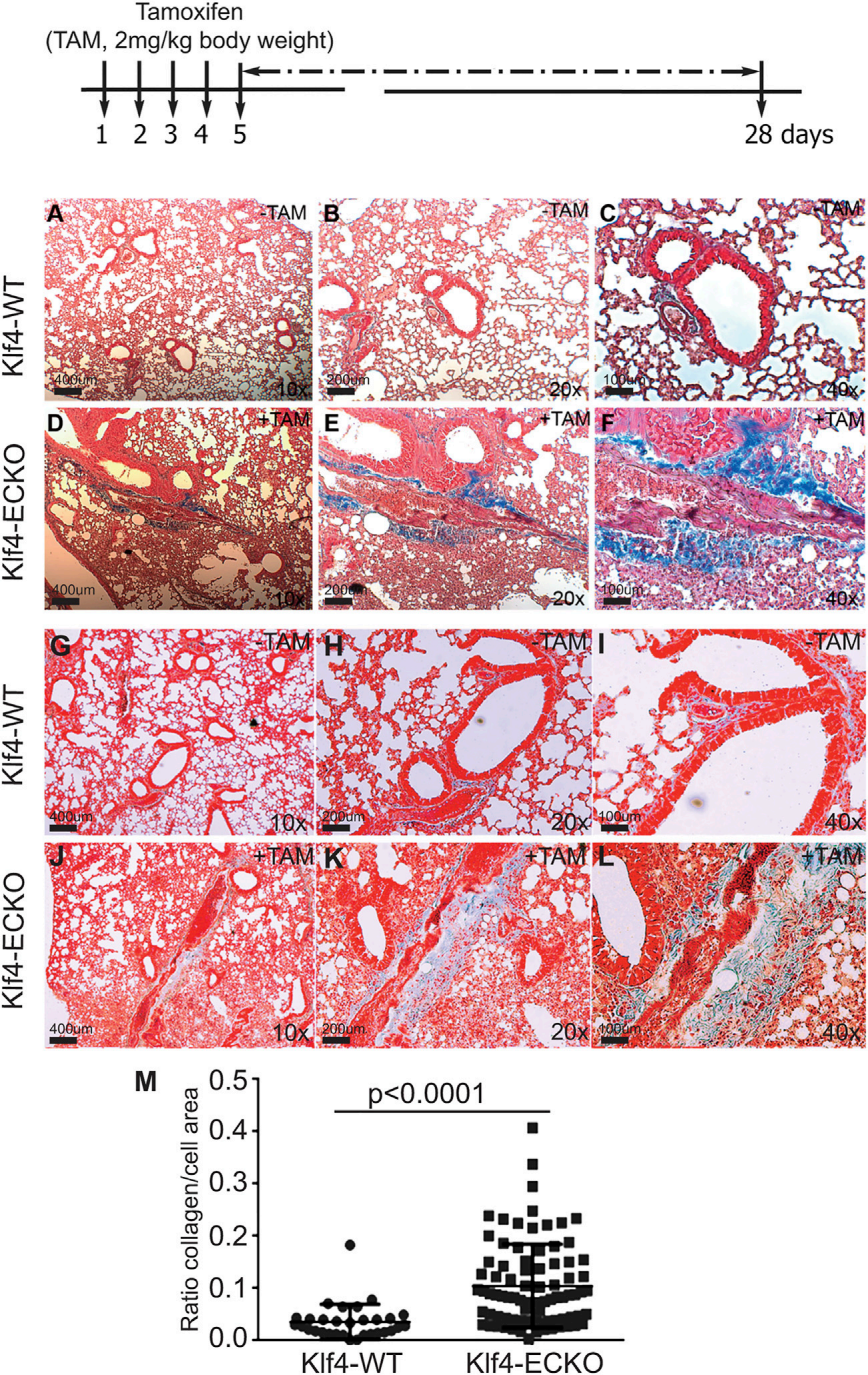
EC-*Klf4* deleted mice harbor appreciable level of fibrosis with alveolar enlargement. Top, strategy and timeline of TAM injection experiments. At the end of experiments (day 26), 8 lungs from control and 8 from *Klf4*-ECKO mice were stained with Masson’s Trichrome. All microvessels in pulmonary cross-sections were imaged on a Zeiss Axiovert microscope at 20X magnification. For blinded measurements, NIH-Image J software was used. Representative images of hematoxylin and eosin (H&E) stained cross-sections of mouse lung showing representative lung architecture. **(A–L)** Normal architecture of alveolar and capillaries in *Klf4*-WT and Klf4-ECKO mice at indicated magnifications (10x, 20x, and 40x). **(M)** Quantification of collagen (blue color) staining Kruskal–Wallis test followed by Dunn’s multiple comparisons to Klf4-WT mice. Data are mean ± S.D. n = 8; *p < 0.0001 vs. Klf4-WT (corn oil).

Recent studies suggest that EndMT-derived myofibroblasts are one of the main drivers of pulmonary fibrosis. To explore the potential connections among the loss of EC-Klf4, EndMT, and lung pathology, tissue sections were subjected to Masson’s trichrome stain. The timeline and strategy are shown at the top (Figure 6). For this experiment, 10 lung lobes were collected from five *Klf4*-WT control and five *Klf4*-ECKO mice. Microscopic examinations were carried-out at 10x, 20x and 40x magnifications (Figures 6A–L) and NIH-Image-J software was used for quantification. *Klf4*-WT lungs showed normal alveolar architecture, while in *Klf4*-ECKO mice the alveolar architecture appeared distorted with an increase in fibroblast density and trichrome+ (blue color) collagen deposition (Figures 6D–F, J–L) which was quantified as shown Figure 6M. Collagen deposition characterized by trichrome staining was consistent with the WB analysis shown in Figure 5C. Therefore, these data indicate that EC-*Klf4* deletion is responsible for lung fibrosis observed in *Klf4*-*ECKO* mice.

### 2.7 Mice harboring EC-*Klf4* deletion show hallmarks of EndMT

**FIGURE 7:**
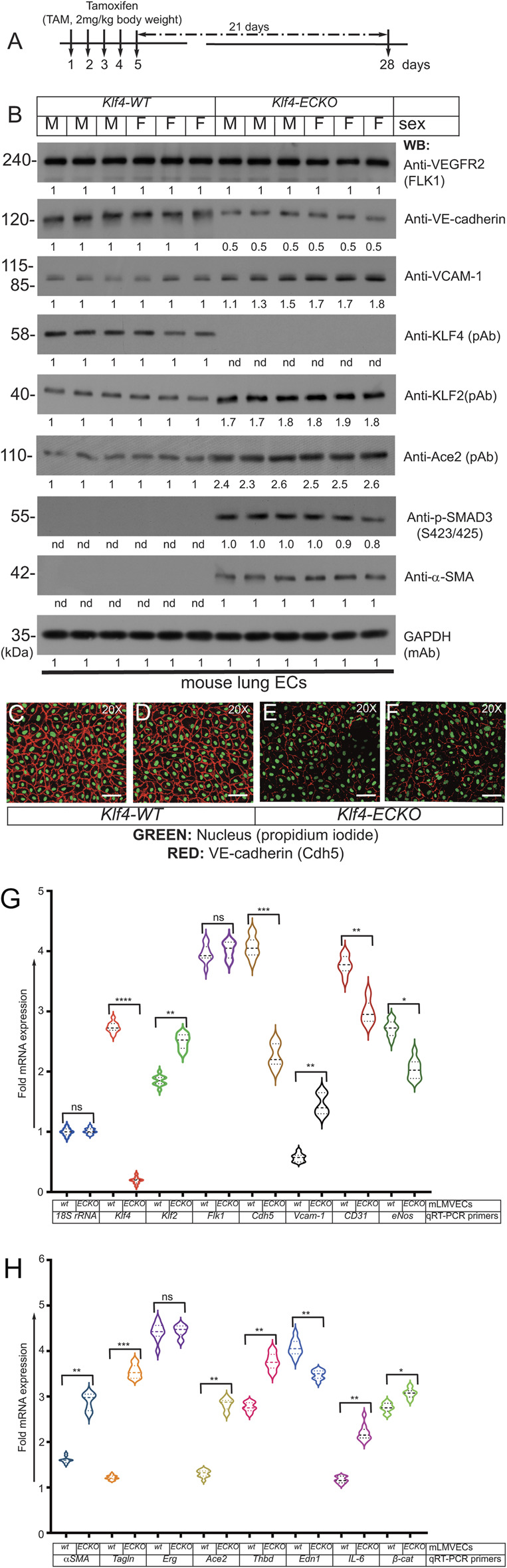
(Continued on next page) EC-Klf4 deleted lung show EndMT. **(A)** Strategy and timeline of experiments. **(B)** Lung microvascular EC protein extracts prepared from *Klf4*-WT (corn oil) mice and *Klf4*-ECKO (+TAM) mice were analyzed by WB with indicated antibodies. VEGFR2 is expressed in all mice with no apparent change in the level of expression. The expression of VE-cadherin in ECs was decreased, while the expression of EC-Klf2, Ace2, and VCAM-1 increased, in ECs of *Klf4*-ECKO mice. We also observed increased phosphorylation of SMAD-3 (S423/425) and acquisition of α-SMA in all ECs that lacked Klf4. There was no change in Gapdh levels. Experiments were repeated at least 3 times. The numbers (quantification) below the WB panels show relative signal intensities. **(C–F)** Representative images of EC characteristics of mouse lung ECs were determined by anti-mouse VE-cadherin (Texas red) staining; nuclear Propidium iodide (green). Magnification, 20×; scale bar, 250 μm.

To further explore the extent of EndMT, primary CD31+/CD45-mLMVECs were isolated from 14 weeks old male and female Klf4-WT and Klf4-ECKO mice. The strategy and timeline of the experiment is shown in Figure 7A. See methods for CD31+/CD45-isolation procedures. Total protein extracts were subjected to WB analyses with anti-VEGFR2/Flk1, anti-VE-cadherin, anti-VCAM-1, anti-Klf4, anti-Klf2, anti-p-Smad3 (S423/425), and anti-α-SMA antibodies (Figure 7B). The expression of VEGFR2/Flk1 did not change in these ECs. Compared to control, there was decreased expression of VE-cadherin in Klf4-ECKO ECs, while EC-Klf2 and VCAM-1 increased. Importantly, the expression of EC-Klf2 and VCAM-1 increased in the Klf4-ECKO cohort. Additionally, we observed increased phosphorylation of Smad3 (S423/425) and acquisition of α-SMA expression in ECs of Klf4-ECKO mice. GAPDH was used to demonstrate equal loading of protein in each lane (Figure 7B). Endothelial cell-specific characteristics of CD31+/CD45-cells isolated from Klf4-WT and Klf4-ECKO was assessed by anti-VE-cadherin staining (Figures 7C–F). Compared to control, ECs isolated from Klf4-ECKO showed decreased VE-cadherin staining by epifluorescence microscopy Figures 7E, F. These data indicate loss of EC-Klf4 is strongly associated with EndMT.

Next, mRNA prepared from *Klf*-WT and *Klf4*-ECKO mLMVECs was analyzed by qRT-PCR assay with mouse gene specific oligonucleotide primers (Table 1). The expression of control *18S rRNA* did not change, while *Klf4* expression decreased significantly in mLMVECs obtained from *Klf4*-ECKO mice (Figure 7G). The expression of *Klf2* increased significantly (*p* < 0.001) in *Klf4*-ECKO group, but Flk1/Vegfr2 did not (Figure 7G). In addition, the expression of several EC-defining genes were decreased in *Klf4*-ECKO mLMVECs, such as *Cdh5* (*VE-cadherin;* ∼ 1.5 fold), as well as *CD31*, *Edn1*, and *eNos* (Figures 7G, H). Importantly, the EndMT markers α*SMA* and *Tagln* (*sm22*) and inflammation markers *Vcam-1* and *IL-6* increased significantly, whereas *Erg* did not **(G,H)** Total mRNAs prepared mLMVECs isolated from Klf4-wt and Klf4-ECKO mice were analyzed by qRT-PCR. 18S rRNA was used as control. Data represent mean ± S.E.M.; ns, not significant; *p < 0.5; **p < 0.01; ***p < 005; ***p < 0.001 versus Klf4-wt as shown. Experiments were repeated 3 times with triplicates. (Figures 7G, H). We also observed significantly increased expression of *Ace2* and *Thbd* in the *Klf4-*ECKO group, as well as a moderate increase of β-catenin expression signifying ongoing EndMT (Figure 7H). These data show that loss of EC-Klf4 is strongly associated with EC dysfunction and EndMT.

**TABLE 1:** Primer Sequences for qRT-PCR.

	Name	Species	Sequence (5’-3’)	Accession#
1	*18S rRNA*	mouse	*(F) 5’-GCA​ATT​ATT​CCC​CAT​GAA​CG-3’*	NR_003278
			*(R) 5’-GGC​CTC​ACT​AAA​CCA​TCC​AA-3’*	
2	*Klf4*	Mouse	*(F) 5'-GAG​GAA​GCG​ATT​CAG​GTA​CAG​AAC-3'*	NM_010637.3
			*(R) 5'-AGG​CTT​ATT​TAC​CTG​GCT​TAG​GTC-3'*	
3	*Klf2*	mouse	*(F) 5'-CCT​CCC​AAA​CTG​TGA​CTG​GTA​T-3’*	NM_008452.2
			*(R) 5'-ATG​CCC​ACC​TGT​CTC​TCT​ATG​T-3’*	
4	*Flk1/Vegfr2*	mouse	*(F) 5’-TGT​GGT​CTC​ACT​ACC​AGT​TAA​AGC-3’*	NM_010612.2
			*(R) 5’-CAT​TCG​ATC​CAG​TTT​CAC​AGA​G-3’*	
5	*Cdh5 (VE-cadherin)*	mouse	*(F) 5’- AGA​TCC​CAG​AAG​AGC​TAA​GAG​GAC-3’*	NM_009868.4
			*(R) 5’- AGA​AAA​GGA​AGA​GTG​AGT​GAC​CAG-3’*	
6	*Vcam-1*	mouse	*(F) 5’-CCC​AGG​TGG​AGG​TCT​ACT​CA-3’*	X67783.1
			*(R) 5’-CAG​GAT​TTT​GGG​AGC​TGG​TA-3’*	
7	*CD31/Pecam-1*	mouse	*(F) 5’-GAG​ACT​CAG​AGG​CGC​TAG​TTA​AT-3’*	NM_008816.2
			*(F) 5’-CTA​ACC​CAG​TGA​TTG​ACA​ACA​GA-3’*	
8	*eNos*	mouse	*(F) 5’-AGA​CCT​CCT​GAG​GAC​AGA​GCT​A-3’*	NM_008713.4
			*(R) 5’-GAA​AAG​CTC​TGG​GTG​CGT​A-3’*	
9	*αSMA*	mouse	*(F) 5′-CCC​CTG​AAG​AGC​ATC​GGA​CA-3′*	NM_007392.3
			*(R) 5’-TGG​CGG​GGA​CAT​TGA​AGG​T-3′*	
10	*Tagln (sm22)*	mouse	*(F) 5’-ATT​GGT​GAA​CAG​CCT​GTA​TCC​T-3’*	NM_011526.5
			*(R) 5’-CTC​CAC​GGT​AGT​TTC​CAT​CG-3’*	
11	*Erg (Ets transcription factor)*	mouse	*(F) 5’- ATC​CTG​GGA​CCG​ACC​AGT​AG-3’*	NM_001302152.1
			*(R) 5’- GTG​ATG​CAG​TTG​GAG​TTG​GAG-3’*	
12	*Ace2*	mouse	*(F) 5’-CTA​CAG​GCC​CTT​CAG​CAA​AG-3’*	AB053182.1
			*(R) 5’-TGC​CCA​GAC​CCT​AGA​GTT​GT-3’*	
13	*Thrombomodulin (Thbd)*	mouse	*(F) 5’-TAG​GGA​AGA​CAC​CAA​GGA​AGA​G-3’*	NM_009378.3
			*(R) 5’-CTT​GCG​CAG​GTG​ACA​GAG​AAG-3’*	
14	*Endothelin-1/Edn1*	mouse	*(F) 5’-CCA​AAG​TAC​CAT​GCA​GAA​AAG​C-3’*	NM_010104.4
			*(R) 5’-CAG​CTT​TCA​ACT​TTG​CAA​CAC​G-3’*	
15	*IL-6*	mouse	*(F) 5’-TAC​CAC​TTC​ACA​AGT​CGG​AGG​C-3’*	NM_001314054
			*(R) 5’-CTG​CAA​GTG​CAT​CAT​CGT​TGT​TC-3’*	
16	*β-Cat (Ctnnb1)*	mouse	*(F) 5’-TGA​CAC​CTC​CCA​AGT​CCT​TT-3’*	NM_007614.3
			*(R) 5’-TTG​CAT​ACT​GCC​CGT​CAA​T-3’*	

F: forward primer; R: reverse primer.

**TABLE 2:** Oligonucleotide primers used for PCR genotyping.

	Primer names	Sequence (5’-3’)
1	Rosa^mT/mG^ (TdTomato/Red)	*(F) 5’-AGC​AAG​GGC​GAG​GAG​GTA​TC-3’*
		*(R) 5’-CCT​TGG​AGC​CGT​ACA​TGA​ACT​GG-3’*
2	Cdh5CreERT2	*(F) 5’-GCG​GTC​TGG​CAG​TAA​AAA​CTA​TC-3’*
		*(R) 5’-GTG​AAA​CAG​CAT​TGC​TGT​CAT​T-3’*
	*Mouse Klf4* specific primers	Sequence (5’-3’)
3	Klf4 exon 1	*(F) 5’-CTG​GGC​CCC​CAC​ATT​AAT​GAG-3’*
4	Klf4 exon 2	*(R) 5’-CGC​TGA​CAG​CCA​TGT​CAG​ACT-3’*
Diagnostic genotyping PCR product sizes		
Genotype	Amplicon size (bp)	
Wild type (wt)	172 bp	
Homozygote (hom)	296 bp	
Heterozygote (het)	296 bp and 172 bp	

### 2.8 EC-Klf4 deletion is associated with alveolar enlargement and increased immune cell adhesion to the endothelium

**FIGURE 8:**
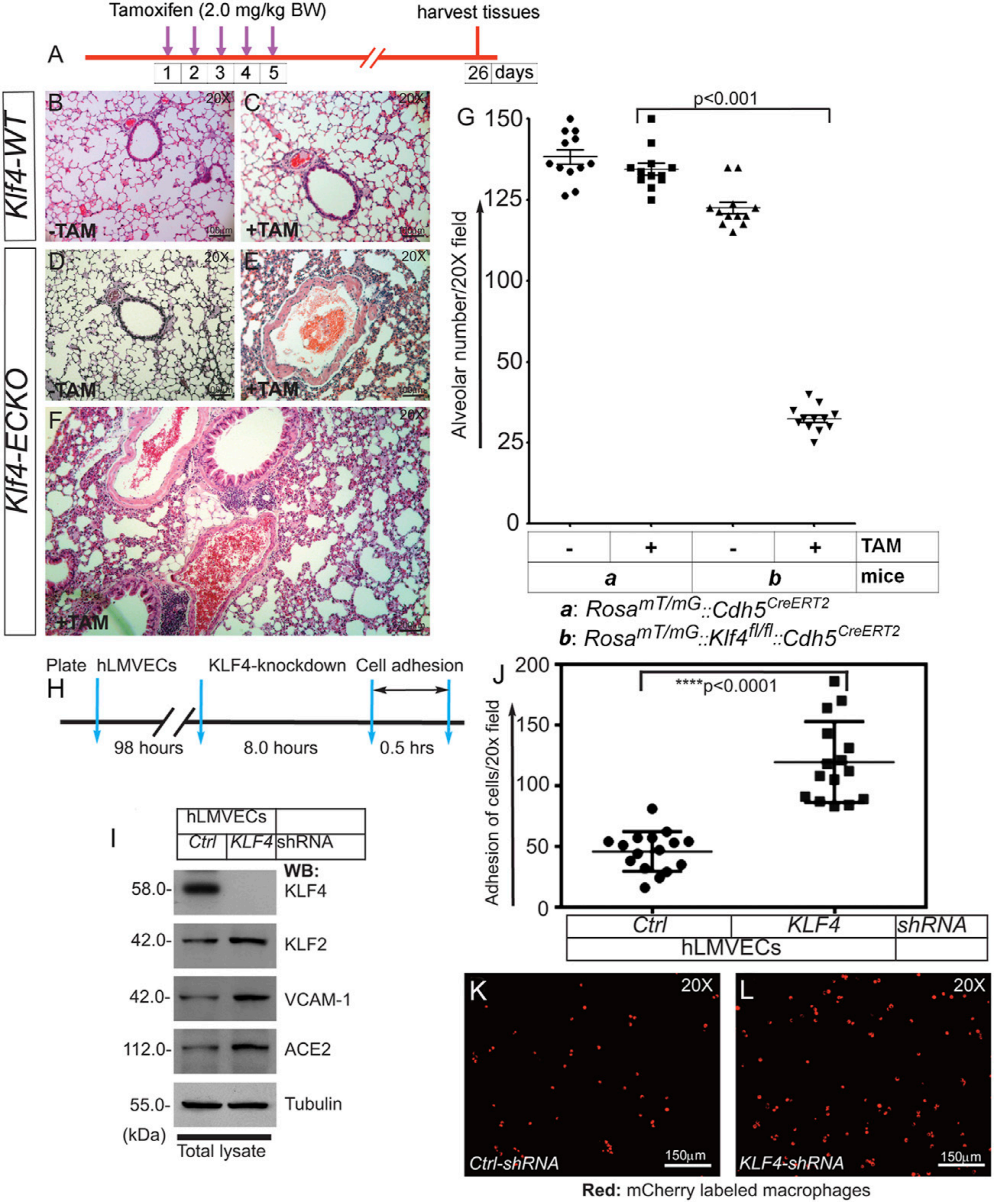
EC-*Klf4* deleted mice harbor decreased number of alveoli and enlarged alveolar structures. **(A)**. Strategy and timeline of experiments. **(B,C)** H&E staining show no change in alveolar structures in control and TAM treated cohorts of Klf4-WT mice. (**D,E)** While there are no changes detectable in Klf4-ECKO (-TAM) cohort, however, there are significant changes accompanied by cellular infiltration and changes in alveolar architecture in a cohort of Klf4-ECKO mice (+ TAM). **(F)** A magnified image of lung pathology in a Klf4-ECKO mice. **(G)** Quantification of alveolar structures. *p* < 0.001 compared with Klf4-WT (+TAM) group. *n* = 12 mice (6 male and 6 female mice) unpaired 2-tailed Student’s t test. Experiments were repeated 2 times. Macrophage adhesion onto confluent human lung microvessel ECs (hLMVECs) monolayer. **(H)** Knockdown, strategy, and timeline of experiment. hLMVECs were treated with *ctrl-shRNA* or *KLF4-shRNA* for 8 hrs. **(I)** Efficiency of knockdown was determined by WB with indicated antibodies. Molecular weights are shown in kDa. **(J)** Next, fluorescent mCherry labeled RAW 264.7 macrophages were allowed to adhere onto hLMVECs. Next, adhesion events were quantified by counting the number of fluorescent mCherry/red macrophages per 20X microscopic field. Quantification macrophage adhesion onto hMLECs in response to KLF4-depletion. *n* = 16–18; ****p < 0.0001 vs. control shRNA. Statistics were carried out using 1-way ANOVA with Newman-Keuls post-hoc test for multiple comparison correction. **(K,L)** Representative confocal images of mCherry (red)-labeled macrophages adhesion onto hLMVECs. Experiments were repeated at least three times.

As we observed extensive EndMT in Klf4-ECKO mouse lung tissues, we next explored whether Klf4 gene deletion was associated with disruption of lung alveolar structures. The timeline and strategy of this experiment is shown in Figure 8A. Cohorts of Klf4-WT and Klf4-ECKO mouse lung tissues were stained with H&E (Figures 8B–F). Detailed morphometric analyses showed no differences in alveolar structures among control mice (Figures 8B–D). In contrast, Klf4-ECKO mice showed widespread alveolar collapse (Figure 8E,F). Quantification revealed a significant decrease in the number of alveoli (Figure 8F,G). We also observed fluid-filled alveoli, sequestration of neutrophils, and degradation of basement membrane in Klf4-ECKO cohorts indicating the presence of vascular inflammation and injury associated with an alveolar pathology (Figures 8E–G). These data indicate that the loss of EC-Klf4 results in altered alveolar structural integrity.

Pro-inflammatory cytokines including TGF-β1, TNF-α, and IL-6 increase expression of cell surface proteins including E-selectin, ICAM, and VCAM-1. These cell surface adhesion molecules recruit and promote firm adhesion of immune cells to injured or activated ECs. Given that Klf4-ECKO was associated with increased expression of VCAM-1 and ACE2, recruitment of inflammatory cells, and extensive EndMT in mouse lung tissues, we next asked the question if EC-KLF4 depletion makes the ECs more adherent to immune cells. To address this question, we performed a cell adhesion assay on KLF4 knockdown ECs (Figures 8H–L). The efficiency of knockdown was determined by immunoblotting (Figure 8I). In the cell adhesion assay, we observed increased attachment of macrophages to monolayer-ECs that received KLF4-shRNA compared to control shRNA treated ECs (Figures 8J–L). This finding indicates that loss of EC-KLF4 increases expression of cell adhesion molecules which bind to counterreceptors such as VLA-4, also known as integrin α_4_β_1_, that are abundantly expressed on the surface of immune cells such as macrophages.

## 3 Discussion

Quiescent ECs crucially maintain lung alveolar homeostasis in adults (Schlereth et al., 2018; Ricard et al., 2021). These cells continuously sense humoral, paracrine, and angiocrine factors and mechanical forces, but are also able to preserve the integrity of the airway microenvironment (Aird, 2007; Eilken and Adams, 2010; Giannotta et al., 2013; Komarova et al., 2017). Our understanding of the molecular mechanisms central to EC quiescence should provide clues to repair mechanisms for EC-dysfunction, normalization of tumor blood vessels, and potential therapies to reverse the course of EndMT. Accordingly, we showed that quiescent ECs express KLF4 and KLF2 proteins which were found bound to each other as a heterodimeric protein complex. However, in the event of a decrease in KLF4 level, KLF2 overcompensated for the loss of KLF4 by upregulating its own expression which led to the disruption of KLF4-mediated quiescent EC phenotype and propagated the emergence of EndMT with alveolar enlargement and fibrosis.

In healthy lung tissues, KLF2 and KLF4 can be detected, however, the expression patterns of KLF4 and KLF2 changed dramatically in diseased lungs. Specifically, the expression of KLF4 decreased, while KLF2 increased significantly in diseased human lung tissues. Quantification showed that KLF4 expression was barely detectable in lungs from patients with emphysema and COPD, while the expression level of KLF2 clearly increased. In addition, we observed increased ACE2 and decreased VE-cadherin in these tissue samples. As TGF-β1 is frequently associated with fibrotic disease, we also addressed TGF-β1 levels in these tissue samples and observed an appreciable increase in the expression of TGF-β1. Since we had access to a very limited number of lung tissue samples, the mechanistic relationship among these proteins and causality of these human diseases is not possible from the data presented. However, increased expression of TGF-β1 strongly corelates with the occurrence fibrosis and thus these data provided the impetus to develop an inducible mouse model test the hypothesis that the oscillations in KLF4 and KLF2 expression might play a central role in the induction of EC dysfunction, EndMT, and fibrosis.

In quiescent ECs showed the presence of mostly monomeric, but also di- and tetrameric forms of KLF4 and KLF2 polypeptide species. These oligomeric complexes were observed in the absence of reducing agent, whereas the addition of reducing agent dissociated the oligomers, thereby producing monomeric forms of KLF4 and KLF2. These results suggest that quiescent ECs harbor mostly monomeric-KLF4 and -KLF2 proteins, but to a lesser extent, dimeric and tetrameric forms can also be found in these cells. One could surmise that there may be more than one pool of KLF4 or KLF2 proteins, e.g., KLF4-monomers KLF4-KLF4 homodimer, and KLF4-KLF4 homotetramer. In addition, KLF4-KLF2 heterodimeric complexes of proteins included p300. A more complex interpretation could be that the quiescent ECs are heterogeneous and the existence of mono-, di- or tetrameric forms of KLF4 and KLF2 might indicate the degree of cellular differentiation or cellular maturation states. In other words, the KLF4-KLF2 complexes might be present in only a subset of quiescent ECs. However, we cannot rule out the possibility that presence of various complexes of KLF4 and KLF2 might also be indicative of different phases of cell cycle. Nevertheless, KLF4 and KLF2 are expressed in quiescent ECs and these two proteins are known to associate directly with p300, a nuclear protein that acts as a histone acetyltransferase (HAT) to transcriptionally regulate targets through chromatin remodeling (Sangwung et al., 2017; Denis et al., 2019; Han et al., 2021). That is, p300 acetylates KLF4 to regulate gene transcription via the modulation of histone acetylation. KLF2, KLF4, and p300 likely form a multimolecular complex in quiescent ECs, consistent with the known ability of transcription factors such as c-Myc to dimerize with Max, Fos with Jun, and β-catenin with TCF4/LEF1. STAT1 homodimerizes with STAT1, and STAT1 heterodimerizes with STAT5. These physical associations are often meaningfully regulated by upstream signaling elements that induce the expression of downstream target genes which could mediate cellular responses such as cell differentiation, proliferation, migration, apoptosis, and survival. However, the mutation, epigenetic modification, or overexpression of transcription factors is associated with pathology. For example, c-Myc and β−catenin mutations are frequently found in many tumors. Importantly, we characterized the amino acid sequence of KLF4 that mediated direct binding to KLF2 to form a heterodimeric proteins complex. These amino acid residues of KLF4 that can form α-helical and coiled-coil secondary structures, and which could form a 3D structure analogous to eukaryotic translation initiation factor 4E (eIF4E)-binding protein 1 (4E-BP1). 4E-BP1 belongs to a family of translation repressor proteins and is a substrate of mTOR signaling cascade, however, it is function in quiescent ECs remains incompletely understood.

What might be the role of p300? One could surmise that by driving acetylation of histones and transcription factors, p300 might epigenetically regulate EC gene expression. In other words, p300 likely provides additional layer of epigenetic control over the endothelial cell-KLF2 and KLF4 gene expression. Nevertheless, the expression analyses demonstrated that KLF4 and KLF2 share overlapping gene targets—about 40% of gene targets are coordinately regulated by both KLF4 and KLF2. The ability of KLF4 and KLF2 to induce expression of a subset of EC gene promoters is not surprising since KLF4 or KLF2 are both known to bind to promoter and enhancer segments harboring 5′-CACCC-3′ or 5′-GGGTG-3′ DNA sequences. However, they can also induce the expression of specific genes in a non-overlapping manner; therefore, most studies have addressed the ability of KLF4 or KLF2 to act alone. While KLF2 and KLF4 can induce the expression of genes independently, no studies have addressed the ability of KLF4 to bind to KLF2 or regulate its expression. Thus, we determined which segment of KLF4 interacts with KLF2.

To clarify the exact relationship between KLF4 and KLF2, we examined the human KLF2-promoter/enhancer. Interestingly, we detected six putative KLF4/KLF2 binding sites in the KLF2-promoter/enhancer segment. This finding could potentially explain why a decrease in KLF4 level might induce the upregulation of KLF2. In control-quiescent ECs, KLF4 bound to the KLF2 promoter, and that a decrease in KLF4 expression was associated with KLF2 autoregulation of its own expression by binding to its own promoter. The ability of KLF4 and KLF2 to bind to the same target sequence was further confirmed by an EMSA experiment. In a converse experiment, KLF2 bound to the same target sequence indicating KLF4 and KLF2 occupy the same exact DNA sequence. This suggests that KLF4 and KLF2 are intrinsically connected and may regulate lung EC gene expression collaboratively and co-operatively.

To address the role of KLF4 in ECs *in vivo,* we generated *Klf4* ^*fl/fl*^ *::Cdh5* ^*CreERT2*^ and *Rosa* ^*mT/mG*^ *::Klf4* ^*fl/fl*^ *::Cdh5* ^*CreERT2*^ mice. We incorporated EC-lineage methodology to label ECs and sorted GFP + ECs for our experiments and EC-specific loss of Klf4 (called *Klf4*-ECKO) was confirmed. Importantly, we detected increased EC-Klf2 expression in *Klf4*-ECKO lung ECs. In *Klf4*-ECKO cells, we also found decreased VE-cadherin expression, but increased Ace, TGF-β1, type-I collagen, and phospho-Smad3 (Ser465/467). A decrease in VE-cadherin signifies the loss of adherens junction mediated cell−cell adhesion, a hallmark of EndMT, while the increase in TGF-β1 and collagen expression are indicative of a pro-fibrotic pathway. Taken together, these data revealed that loss of EC-Klf4 is associated with increased Klf2 expression and EndMT. To explore further the mechanistic link between loss of EC-Klf4, EndMT, and lung pathology, tissue sections were analyzed by Masson’s trichrome staining which revealed damaged and distorted alveolar architecture in *Klf4*-ECKO mice. There were also indications of immune cell infiltration, enlarged alveoli, and deposition of collagens. Together, these data strongly indicated that the loss of EC-Klf4 is associated with lung disease.

Furthermore, *Klf4*-ECKO mice showed decreased VE-cadherin, but increased Klf2, VCAM-1, Ace2, phospho-Smad3 (S423/425), as well as increased αSMA levels**,** decreased VE-cadherin at cell-cell junctions of endothelial cells lining lung microvessels. In addition, Expression of *Klf2*, *Vcam-1*, *IL-6*, *α-SMA*, *Tagln*, *Thbd*, and *β-catenin increased*, while *Klf4*, *Cdh5*, *CD31*, and *eNos* mRNA decreased, signifying EC-dysfunction and ongoing EndMT. Thus, these data reveal the loss of EC-Klf4 is strongly associated with EndMT. In addition, we detected neutrophils and degradation of basement membrane in *Klf4-*ECKO cohorts indicating the presence of EC dysfunction and alveolar damage. Therefore, these data illustrate that the loss of EC-Klf4 is incompatible with the normal alveolar structural integrity.

It is increasingly clear that immune cells adhere to the endothelium in response to inflammation, injury, and regenerative processes. As Klf4-deficient ECs showed increased expression of Klf2, VCAM-1, ACE2, and IL-6, we addressed whether the endothelium loses its anti-inflammatory and anti-adhesive properties. It is unclear why after induction of KLF2, there was no increased expression of Flk1 or vWF in the lung ECs. It remains possible, however, that in other vascular bed, e.g., kidney and heart, the expression of Flk1 and vWF could have increased. To address this possibility directly, RNA-seq experiments could be carried out in the future, to explore the organ-specific EC-KLF2 and -KLF4 functions. Nevertheless, KLF4 knockdown increased adhesion of macrophages onto monolayer ECs. Specifically, we observed increased attachment of macrophages to EC monolayers that received KLF4-shRNA. This indicated that the loss of EC-KLF4 promotes the expression of cell adhesion molecules such as VCAM-1, which then can bind to a counter-receptor such as VLA-4 (aka α_4_β_1_ integrin) expressed on monocytes/macrophages. Both KLF2 and KLF4 are downstream regulators of laminar sheer stress (LSS), thus, the loss of KLF4 could have altered the LSS regulated endothelial homeostatic gene regulation. Together, these findings are consistent with the known function of KLF4 to act as an anti-inflammatory and atheroprotective transcription factor (Kuo et al., 1997; Sangwung et al., 2017).

The loss of Klf4 and upregulation of Klf2 in the lung ECs was accompanied by alveolar enlargement, a key hallmark of chronic inflammatory lung disease. Increased accumulation of immune cells and elaboration of inflammatory cytokines in the lung, can lead to destruction of the basement membrane and compromised alveolar structural integrity. Thus, the loss of Klf4 in quiescent ECs is primarily responsible for endothelial dysfunction, vascular leakage, and EndMT, and secondarily promotes neutrophil and macrophage recruitment in the lung that gave rise to alveolar enlargement and fibrosis.

## 4 Conclusion

**FIGURE 9:**
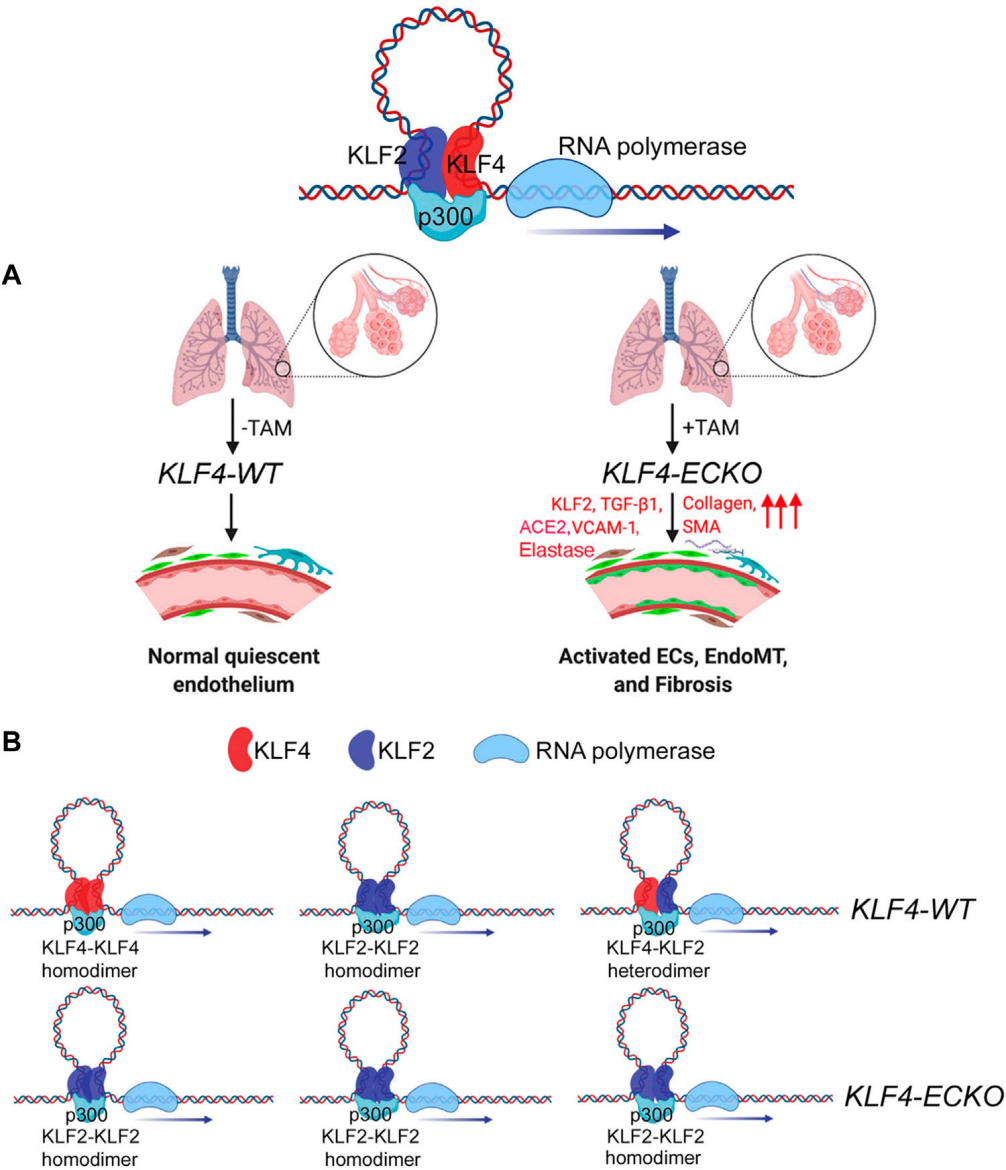
KLF4 and KLF2 in mediating EndMT in the lung. **(A)** In lung capillaries that surround alveolae, quiescent ECs express both monomeric and heterodimeric KLF2-KLF4 protein complexes. The loss of KLF4 triggers an adaptive response by upregulating KLF2 expression, which in turn induces the expression of collagens, VCAM-1, SMA, and SM22 resulting in the recruitment of immune cells and transition of quiescent ECs into EndMT. **(B)** Homodimeric and heterodimeric KLF2 and KLF4 protein complexes in KLF4-WT and KLF4-ECKO mice. The above images were obtained through online Biorender.com graphical image bank and templates.

KLF4 and KLF2 are highly expressed in quiescent ECs. However, EC-specific inducible *Klf4*-deletion induced irreversible EC activation and EndMT. Loss of KLF4 was accompanied by an increase in KLF2 expression, increased VCAM-1, ACE2, and IL-6 expression, and recruitment of circulating immune cells. An emerging hypothesis that stems from these observations is that the loss of quiescent EC-KLF4 is likely pathogenic, therefore normalization of lung microvessel KLF4 levels in patients with chronic lung disease might reverse the course of EndMT and restore the EC quiescence (see graphical abstract, Figure 9).

## 5 Methods and materials

Antibodies information is provided in a table

### 5.1 Cell-Molecular Biology and Biochemical Reagents

TRIzol (15596018), and Lipofectamine™ 2000 (11,668–019) were purchased from Invitrogen (Carlsbad, CA). Tamoxifen (J6350903, Alfa Aesar, Ward Hill, MA; T5648-1G, Sigma) stock was dissolved in corn oil (C8267, Sigma), filtered with a sterile 0.22 μM filter, and stored at −20°C in a dark sealed container. It was warmed to room temperature prior to use. Lentivirus encoding control-shRNA and KLF4-shRNA knockdown vectors were purchased from Open Biosystems, Inc. (RHS3979-201737586, RHS3979-201737587, RHS3979-201737588, RHS3979-201737589, RHS3979-201742512, Huntsville, AL, United States).

### 5.2 Cell culture and media

These methods have been previously described (Baruah et al., 2020; Chatterjee et al., 2016; Humtsoe et al., 2003; Kohler et al., 2011; Kohler et al., 2013). All primary endothelial cells (ECs) were cultured up to 3-5 passages in EndoGro^TM^ Basal Medium with EndoGro^TM^ VEGF Supplement Kit (SCME-BM; SCME002-S; millipore) on 0.2% gelatin (G-2625, Sigma-Aldrich) in PBS-coated plates. Amphopack 293T virus packaging cells and murine RAW 264.7 macrophage cells were cultured in Dulbecco’s minimum essential media (DMEM) (01–055-1, Biological Industries) supplemented with L-glutamine (25–005-CI; Corning) and 10% fetal bovine serum (F2442; Sigma-Aldrich).

### 5.3 Co-Immunoprecipitation (Co-IP) Experiments

For Co-IP, adherent primary hLMVECs at 100% confluency were washed three times with cold 1x PBS, pH 7.4, followed by an incubation in 1x RIPA buffer on ice as previously described (Baruah et al., 2020; Chatterjee et al., 2016; Kohler et al., 2011; 2013). For co-IP experiments, mouse IgG agarose beads and Protein-G Sepharose beads were washed three times with lysis buffer, and subsequently resuspended in PBS each to make 50% IgG agarose beads and 50% Protein-G Sepharose bead solutions, respectively. To reduce the amount of proteins that bind nonspecifically, the cell lysates were incubated with 30 μl of mouse IgG agarose beads on a rotator for 1 h at 4°C. Beads/slurry were centrifuged at 5,000 g for 30 s at 4°C and supernatant protein lysate transferred to a new 1.5 ml centrifuge tube. Immunocomplexes were washed 5 times with 1XTris, pH 7.5 buffer. Thereafter, each sample was diluted with 2X reducing sample buffer containing (5% β-mercaptoethanol) and boiled in a water bath for 5 min, and resolved by 10% SDS-PAGE, then transferred to as previously described (Baruah et al., 2017; Baruah et al., 2020; Chatterjee et al., 2016; Humtsoe et al., 2003; Kohler et al., 2011; Kohler et al., 2013).

### 5.4 Human lung disease tissues and immunohistochemistry (IHC), and microscopy

The Lung Tissue Research Consortium (LTRC) at the National Health Institutes (NIH) has a collection of lung disease tissues. The program enrolled donors who underwent lung surgery, and blood and extensive phenotypic data were collected from the prospective donors, thereafter the surgical waste tissues are processed for research use. Most donor subjects harbored interstitial fibrotic lung disease, emphysema or COPD. Phenotypic data include clinical and pathological diagnoses, chest computed tomography (CT) scanning images, pulmonary function tests, exposure and symptom questionnaires, and exercise tests. Tissue samples used in this study were obtained from the LTRC for limited research use for biochemical and histological analyses. Portions of lung tissue samples were fixed in zinc formalin (10%) solution and embedded in paraffin as previously described (Baruah et al., 2020; Chatterjee et al., 2016; Kohler et al., 2011; Kohler et al., 2013). At least twelve to fifteen serial sections (6–8 µm thin) were prepared of each paraffin embedded organ. After deparaffinization, dehydration, and rehydration, the sections were permeabilized in 0.5% Triton X-100 in 1x PBS for 30 min and washed in 1X PBS. The sections were washed 3 times for 5 min in 1x PBS and incubated with secondary antibody (1:200, 2.5% BSA in 1x PBS) for 1 h at RT in a humidified chamber, washed 3 times in 1x PBS. The slides were mounted using DAPI anti-fade gold reagent. Thereafter sections were examined under the Olympus BX51/IX70 fluorescence microscope (Olympus, Tokyo, Japan) and quantified using National Institute Health (NIH)-ImageJ software (Bethesda, MD, United States).

ECs up to passage 5 were seeded at 50–60% confluency on sterile glass coverslips coated with 0.02% gelatin in 1x PBS, pH 7.4. ECs were cultured for 4–5 days as described previously (Baruah et al., 2020; Chatterjee et al., 2016; Humtsoe et al., 2003; Kohler et al., 2011; Kohler et al., 2013). Cells fixed in 4% PFA, washed in 1XPBS, permeabilized in 0.5% Triton X-100 (Fischer Scientific) in 1x PBS for 30 min at room temperature, followed by 3 washings in 1x PBS. Coverslips were incubated in 0.5% BSA, thereafter primary antibody applied for 1 h (1:200 dilution with 0.5% BSA). After 1x PBS wash, secondary antibody (1:500 dilution with 0.5% BSA) was applied for 30 min. Coverslips were washed in 1x PBS and then mounted with ProLong® Diamond Antifade Mounting reagent with DAPI (P36962, Invitrogen). Digital microscopic images were acquired using Olympus BX51/IX70 Fluorescence microscope, the EVOS FL Cell Imaging System (InVitrogen), or Zeiss LSM 880 Confocal microscope saved as TIFF and CZI. Multiple panel figures were assembled using QuarkXpress 10.0 software. Final images were saves as EPS or TIFF documents.

### 5.5 Production of pLentivirus and shRNA-KLF4 knockdown

To amplify DNAs, we transformed *E. coli* (DH5α) with the five pLentivirus encoding shRNAs targeting *KLF4* gene and a scrambled shRNA control construct as previously described (Baruah et al., 2020; Chatterjee et al., 2016; Humtsoe et al., 2003; Kohler et al., 2011). To generate viral particles encoding the shRNAs, amphopack-293T cells were transfected with lentivirus encoding shRNA constructs using Lipofectamine as described previously (Baruah et al., 2020; Kohler et al., 2011). After 24 h, transfected cells were fed with fresh media. After 36–48 h, cells were detached and replated into 1:3 ratio in media containing 7 mg/ml puromycin (A11138-03, ThermoFischer Scientific). Puromycin media was changed every 3–4 days to remove dead cells. After 12–14 days, surviving cells/colonies were pooled and replated onto dishes. These cells were expanded into 1:3 ratio. After 3 days, one dish of cells was frozen, and two dishes were passaged for virus supernatant generation. Viral particles were collected in complete EC media and used immediately. The efficiency of knockdown was determined by RT-PCR and Western blot as previously described (Baruah et al., 2020; Kohler et al., 2011; Humtsoe et al., 2003).

### 5.6 Macrophage cell adhesion assay

Cell adhesion assay has been previously described (Humtsoe et al., 2003). ECs were grown to 60–80 percent confluency on 6 well plates coated with 0.02% gelatin in 1x PBS, pH 7.4 as previously described. ECs were treated with either control or KLF4 stealth siRNA for 6 h, and 24 h later were treated with either PBS or LPS (20 ug/ml) for 6 h. RAW 264.7 macrophages were labeled with mCherry using CellTracker™ Red CMTPX (C34552, Invitrogen) protocol. Fluorescently labeled RAW 264.7 macrophages were allowed to adhere to ECs for 0.5 h as previously described (Humtsoe et al., 2003). ECs were washed to remove non-adherent cells and macrophage adherence was quantified by counting the number of fluorescent red macrophages per field using either the LSM 880 confocal or the EVOS FL Cell Imaging System.

### 5.7 Quantitative (q)-reverse transcribed (RT)-PCR

To isolate total RNA, cells were grown to confluency and RNA samples were collected using either TRIzol or RNAzol RT reagent as previously described (Kohler et al., 2013; Chatterjee et al., 2016; Baruah et al., 2017). The mRNAs were resuspended in 100 μl of RNAse free water and incubated at 55°C for 10 min, quantified, and stored at -80°C or used immediately. Automated qRT-PCR assay was carried out by a QuantStudio 7 Flex (Applied Biosystems) using SYBR green master mix (4367659, Applied Biosystems) with forward and reverse primers (Table 1) custom made through IDT (Coralville, IO). Data was analyzed using Δ-Δ-CT values normalized to housekeeping genes such as 18S-rRNA or GAPDH using Expression-Suite software (ThermoFisher Scientific).

### 5.8 Generation of Klf4 genetically engineered mouse model (GEMM)

Mice were housed in a pathogen-free vivarium and handled in accordance with standard use procedures, animal welfare regulations, and the National Institutes of Health (NIH) Guide for the Care and Use of Laboratory Animals. All experimental procedures and husbandry have been approved by the University of Illinois at Chicago (UIC) Institutional Animal Care and Use Committee (IACUC). We procured Klf4^fl/wt^ mice on a C57BL background (Dr. Klaus Kaestner, University of Pennsylvania, Philadelphia, PA) (Katz et al., 2005), obtained from Mutant Mouse Resource & Research Centers (MMRRC) through an institutional Material Transfer Agreement (MTA). Introns 1 and 3 of the *Klf4* gene have *loxP* sites to create the *floxed* allele. These mice were crossbred to produce a generation of offspring such that a percentage of these mice were homozygous for the *Klf4* ^*fl/fl*^ mutant genotype. Our study used both male and female mice ranging from 12 to 14 weeks of age. For mouse experiments, no randomization was done, or no animals were excluded from this study. *Gt(Rosa)* ^*26Sortm4(ACTB-tdTomato,-EGFP)Luo/J*^ (stock#007576, hereafter called *Rosa* ^*mT/mG*^) mice were bought from the Jackson Laboratory (Bar Harbor, ME) (Baruah et al., 2020; Gong et al., 2015) and the *tg. Cdh5*(*PAC*)^*CreERT2*^ (also known as *Tg:VE-cadherin-*(*PAC*)^*CreERT2*^, and hereafter called *Cdh5* ^*CreERT2*^) transgenic mice were obtained from Ralph H. Adams (Wang et al., 2010) through an MTA from the United Kingdom *Cancer* Research (London, United Kingdom). Following TAM (2.0 mg/kg body weight, BW) administration for each of five consecutive days, all ECs (expressing EC-specific *Cdh5* ^*CreERT2*^) turn green due to mG expression, while non-ECs fluoresce tomato (red) due to baseline expression of mT. The transgenic *Cdh5* ^*CreERT2*^ driver line served as a proxy for EC specificity (Baruah et al., 2020). After a series of breeding and backcrossing regimens, *Klf4* ^*fl/fl*^ *::Cdh5* ^*CreERT2*^, *Rosa* ^*mT/mG*^ *::Cdh5* ^*CreERT2*^ and *Rosa* ^*mT/mG*^ *::Klf4* ^*fl/fl*^ *::Cdh5* ^*CreERT2*^ GEMM were produced.

### 5.9 Genotyping of GEMM

Mouse tails were snipped (2 mm) and the ears tagged aseptically as previously described (Chatterjee et al., 2016). DNAs were isolated using Phenol:Chloroform:Isoamyl alcohol method (Kohler et al., 2013). To induce deletion of EC-*Klf4* in a timed-manner, we injected 12–14 weeks old adult mice with 2.0 mg/kg body weight TAM once every day intraperitoneally (i.p.) for 5 consecutive days. After treatment with TAM, we waited for 21 and 28 days so that all TAM were metabolized and excreted, thereafter mice were humanely sacrificed, different organs harvested, fixed, sectioned, and remaining tissues were processed for EC isolation, RNA and DNA extractions, and protein lysate preparation as previously described (Kohler et al., 2013; Chatterjee et al., 2016).

### 5.10 Statistics

Data were calculated and expressed as mean ± S.D. and values plotted with Microsoft Excel or using GraphPad Prism 6 (GraphPad Software, La Jolla, CA, United States). For comparisons between two groups, an unpaired Student’s t-test was performed, and analysis of variance (ANOVA), followed by Tukey’s, Sidak’s, or Dunnett’s test for multiple comparisons. Number of experiments or number of animals represents biological replicates and a *p* value of <0.05 was considered significant. qRT-PCR data expression was calculated using ΔΔCT values normalized against both 18s RNA and GAPDH of control samples.

## Data Availability

The original contributions presented in the study are included in the article/Supplementary Material, further inquiries can be directed to the corresponding author.

## Supplementary material

Supplementary Figure S1
*KLF4* and *KLF2* transcripts in human tissues. Screen grabs of RNA-seq analyses show *Klf4* (www.ncbi.nlm.nih.gov/gene/9314) and *KLF2* (www.ncbi.nlm.nih.gov/gene/10365) transcripts in normal human tissues, and abundantly found in the lung.

Supplementary Figure S2 Analysis of KLF4, KLF2, and TGF-β1 in human lung inflammatory disease samples. Control healthy donors C&D. Lung tissues obtained from emphysema and COPD patients were stained with anti-KLF2 or KLF4. Representative photomicrographs of remodeled pulmonary vessels, decreased alveolar counts in COPD patients, compared with healthy vessels in donors are shown. **(E)** Quantification of KLF2 and KLF4 positive nuclei (dark brown). Data represents mean ± S.D. and p values and “n” are as shown. **(F)** The cell lysates were subjected to Western blotting (WB) with antibodies against KLF4, KLF2, TGF-β1 and GAPDH. GAPDH was used as a control for equal protein loading. The numbers below each panel represent signal intensities relative to GAPDH. Experiments were repeated at three times. *n* = number of samples. P < 0.05 is considered statistically significant. Scale bar and magnifications areas are as shown.

Supplementary Figure S3 Human lung disease harbor microscopic EndMT. A thin section (6-7μ) of emphysema tissue section was immunostained with anti-CD31 (green) and anti-α-SMA (red) antibodies and analyzed by epifluorescence microscopy at indicated magnification. Blue color indicates nuclear staining (DAPI). Magnifications and scale bars are as shown. The brightness and contrast of this image was not adjusted. Quantification was not performed due to limited sample size.

Supplementary Figure S4 KLF4(91-117aa) 2D and 3D structure computational analysis. Amino acid residues -NDLLDLDFILSNSLTHPPESVAATVS- was submitted for Iterative Threading ASSEmbly Refinement (I-TASSER) (https://zhanglab.dcmb.med.umich.edu/I-TASSER/). I-TASSER is a protein structure prediction and structure-based function annotation tool. It identifies and retrieves structural templates from the Protein Database (PDB) by multiple threading method LOMETS, with full-length atomic models constructed by iterative template-based fragment assembly simulations.

Supplementary Figure S5 Generation of *Rosa* ^*mT/mG*^ *::Klf4* ^*fl/fl*^ *::Cdh5* ^*CreERT2*^ mice line. A The timeline and TAM treatment scheme; Representative ethidium-bromide stained agarose gel images of PCR genotyping. Genomic DNA of *wild‐type (WT) Klf4, floxed (fl) Klf4, Cdh5* ^*CreERT2*^ (*Cre*)*, and Rosa* ^*mT/mG*^ mice were amplified by PCR Table 2. The WT band is 464 bp, the floxed Klf4 product is 618 bp, and the *Cdh5* ^*CreERT2*^ product is 934 bp. *MW, ladder*. **F** EC extracts were prepared from indicated mouse lung tissues and analyzed by WB with indicated antibodies. Molecular weights are given in kiloDalton (kDa). The numbers below each WB panel indicate signal quantification. Experiments were performed at least 3 times.
