# Supplementary material for: A requirement for Krüppel Like Factor‐4 in the maintenance of endothelial cell quiescence
Source: Front Cell Dev Biol. 2022 Nov 8;10:1003028. doi: 10.3389/fcell.2022.1003028 (PMC9679496; doi:10.3389/fcell.2022.1003028)
Supplement: Supplementary file 1 [file DataSheet2.PDF]

KEY RESOURCES TABLE

REAGENT OR RESOURCE			**CATALOG# Or RRID#**
**Antibodies**	**Dilution**	**SOURCE**	
Goat anti-mouse/rat CD31/PECAM-1	1:25 (staining)	R&D Systems	AF3628
Goat anti-mouse/rat CD31/PECAM-1	1:100 (flow)	R&D Systems	AF3628
Mouse monoclonal anti-KLF4	2 μg/ml (WB)	Rockland	200-301-CE9
Rabbit Lamin-B1	2 μg/ml (WB)	Rockland	600-401-P62
Mouse anti-α-smooth muscle actin	2 μg/ml (WB)	Sigma-Aldrich	A2547
Rat ant-CD31 (MEC7.46)	1:25 (staining)	Abcam	Ab7388
Rat ant-CD31 (MEC7.46)	1:200 (flow)	Abcam	Ab7388
Rabbit polyclonal anti-TAGLN/Transgelin	1:25 (staining)	Abcam	ab14106
Sheep anti-von Willebrand Factor (vWF)	1:25 (staining)	Abcam	ab11713
Rabbit anti-Collagen I	2 mg/ml (WB)	Abcam	ab34710
Anti-sheep IgG-Alexa Fluor 488	1:200	Abcam	ab150177
Anti-rabbit IgG-Alexa Fluor 647	1:200	Abcam	ab150063
Mouse monoclonal anti-Klf4 (F8)	2 μg/ml (WB)	Santa Cruz	166238
Rabbit anti-KLF4 (H-180)	2 μg/ml (WB)	Santa Cruz	sc-20691
Rabbit anti-KLF2 (H-60)	2 μg/ml (WB)	Santa Cruz	sc-28675
Mouse monoclonal anti-TGF-β1	2 μg/ml (WB)	Santa Cruz	sc130348
Mouse monoclonal anti-p300 (NM11)	2 μg/ml (WB)	Santa Cruz	sc-32244
Mouse anti-VEGFR2/FLK1 (A3)	2 μg/ml (WB)	Santa Cruz	sc-6251
Mouse monoclonal anti-Endoglin	1:25 (staining)	Santa Cruz	sc-376381
Monoclonal anti-Endoglin (P3D1)	2 μg/ml (WB)	Santa Cruz	sc-18838
Mouse anti-VE-cadherin (Cdh5/CD144)	1 μg/ml (WB)	Santa Cruz	sc-9989
Rat anti-mouse CD31	1:200 (flow)	BD Biosciences	550274
Rabbit anti-von Willebrand factor (vWF)	1:40 (staining)	EMD Millipore	AB7356
Rabbit anti-GAPDH	1 μg/ml (WB)	Cell Signaling	5174
Rabbit anti-VEGFR2/FLK1 (55B11)	2 μg/ml (WB)	Cell Signaling	2479
Rabbit anti-eNOS (D9A5L)	2 μg/ml (WB)	Cell Signaling	32027
Rabbit anti-α-SMA (D4K9N)	2 μg/ml (WB)	Cell Signaling	19245S
Rabbit anti-P-Smad2 (S465/467)	2 μg/ml (WB)	Cell Signaling	3108S
Rabbit anti-mouse VCAM-1 (D8U5V)	2 μg/ml (WB)	Cell Signaling	39036
Rabbit anti-human VCAM-1 (E1E8X)	2 μg/ml (WB)	Cell Signaling	13662
Rabbit anti-FLAG (M2) (D6W5B)	2 μg/ml (WB)	Cell Signaling	14793
Rabbit anti-FLAG (M2) (D6W5B)	2 μg/IP (co-IP)	Cell Signaling	14793
Rat anti-mouse VE-cadherin (Cdh5/CD144)	2 μg/ml (WB)	Thermo-Fisher	14-1441-82
Rat anti-mouse VE-cadherin (Cdh5/CD144)	1:100 (flow)	Thermo-Fisher	14-1441-82
Anti-KLF4 antibody (11880-1-AP)	2 μg/ml (WB)	Thermo-Fisher	PA5-27440
Anti-KLF2 antibody	2 μg/ml (WB)	Thermo-Fisher	PA5-40591
Anti-ACE2 Monoclonal Antibody (CL4035)	2 μg/ml (WB)	Thermo-Fisher	MA5-31395
Rabbit anti-KLF4	3 μg/ml	Thermo-Fisher	PA5-35303
Donkey anti-rabbit Alexa Flour-594	1:200	Thermo-Fisher	AB-141637
Chicken anti-rabbit Alexa Fluor-647	1:200	Thermo-Fisher	AB-2535861
Goat anti-mouse Alexa Flour-488	1:200	Thermo-Fisher	AB-2534069

Mouse anti-human KLF4 antibody	2μg/IP	Novus	H00009314-M01
Anti-rabbit IgG-TRITC	1:200	Novus	NBP1-75270
Anti-mouse IgG-DyLight 594	1:200	Novus	NBP1-75563
Mouse anti-Tubulin (AA10)	1 μg/ml (WB)	BioLegend	657402
Anti-mouse CD11b (clone M1/70)	1:200 (flow)	BioLegend	101206
Anti-LY-6G (clone 1A8)	1:200 (flow)	BioLegend	127610
Mouse anti-GST antibody (P1A12)	1 μg/ml (WB)	BioLegend	640802
**Mice and Animals**	**Sex**	**SUPPLIER**	**STOCK#**
Klf4$^{wt/fl}$	Breeder pair	MMRRC	29877
Gt(Rosa)$^{26Sortm4(ACTB\text{-}tdTomato,\text{-}EGFP)Luo/J}$	Breeder pair	JaxLab	007576
tg.Cdh5(PAC)^{CreERT2}	Breeder pair	UK Cancer Res	---
**Cells**	**Abbreviation**	**SUPPLIER**	**CAT#**
Human umbilical vein endothelial cells	HUVECs	LONZA	C2519A
Human lung microvessel endothelial cell	hLMVECs	PromoCell	C-12281
RAW 264.7 macrophage cells	Macrophages	Kostandin Pajcini	gift
HEK293T Packaging cell line	Phenix-Ampho	Genscript	T98134
**Chemicals, Media and Reagents**		**SUPPLIER**	**CAT#**
Lenti-X Concentrator		Clontech/TAKARA	631321
Polybrene infection reagent	Polybrene	Merck	TR-1003-G
Tamoxifen	TAM	Sigma/Millipore	T5648
Corn oil		Sigma/Millipore	C8267
Dulbecco's Modified Eagle Medium	DMEM	Sigma/Millipore	FG4815-BC
Endothelial Cell Media kit	EndoGRO	Sigma/Millipore	SCME004
Elastase assay kit		Sigma/Millipore	MAK246-1KT
Fetal Bovine Serum	FBS	Sigma/Millipore	TMS-013-B
L-Glutamine	L-Glu	Sigma/Millipore	K0282-BC
Antibiotic antimycotic solution (100x)	PenStrep	Sigma/Millipore	A5955-100ML
Gelatin from porcine skin	Gelatin	Sigma/Millipore	G2500-100G
Bis-Acrylamide solution (1:30)	PAGE	BioRad	1610156
Tetramethylethylenediamine	TEMED	BioRad	161-0801
Alpha-1-Anti-Trypsin (A1AT)	Prolastin	Grifolis	--
Microbeads coupled mouse CD45 antibody	CD45-beads	Miltenyi Biotech	130-052-301
Microbeads coupled mouse CD31 antibody	CD31-beads	Miltenyi Biotech	130-097-418
Secrete-Pair kit		GeneCopoeia	LF031
SYBR Green master mix		Applied Biosystm	4385612
Apoptosis-detection kit APC		eBiosciences	88-8007-72
ELISA kit, IL-1β		ThermoFisher	BMS6002
ELISA kit, TNFα		ThermoFisher	BMS607-3
ELISA kit, IL-6		ThermoFisher	KMC0061
Puromycin	6μg/ml	ThermoFisher	A11138-03
Prolonged Gold DAPI with antifade		ThermoFisher	P36962

Plasmids and vectors		SUPPLIER	CAT#
pLNCX2 (lentivirus)	DNAs	Takara	631503
Klf4-shRNA	Lentivirus	OpenBiosystems	---
pLenti-C-Myc-DDK (PS100064)	Lentivirus	Origin	---
Human KLF4 cDNA (NM_004235)	Lentivirus	Origin	RC206691L1
Human KLF2 cDNA ((NM_016270)	Lentivirus	Origin	RC210042L1
pEZX-LvG04	Virus vector	GeneCopoeia	custom
pLNCX2, pLNCX2-shRNAs	DNAs	Genscript	custom
KLF4-WT and mutant-deletion constructs	DNAs	Genscript	custom
GST-KLF4 fusion proteins constructs	DNAs	Genscript	custom
